# Changes in nitric oxide inhibitors and mortality in critically ill patients: a cohort study

**DOI:** 10.1186/s13613-024-01362-7

**Published:** 2024-08-27

**Authors:** Karoline Myglegård Mortensen, Theis Skovsgaard Itenov, Jakob Stensballe, Thore Hillig, Claus Antonio Juel Jensen, Martin Schønemann-Lund, Morten Heiberg Bestle

**Affiliations:** 1 Department of Anesthesiology and Intensive Care, Copenhagen University Hospital – North Zealand, Hilleroed, Denmark; 2 Department of Anesthesiology and Intensive Care, Bispebjerg and Frederiksberg Hospitals, Copenhagen, Denmark; 3 Section for Transfusion Medicine, Capital Region Blood Bank, Rigshospitalet, Copenhagen, Denmark; 4 Department of Anesthesiology, Surgery and Trauma Center, Copenhagen University Hospital – Rigshospitalet, Copenhagen, Denmark; 5 Department of Clinical Biochemistry, Copenhagen University Hospital – North Zealand, Hilleroed, Denmark; 6 Department of Clinical Medicine, University of Copenhagen, Copenhagen, Denmark

**Keywords:** Nitric oxide, Asymmetric dimethylarginine, Symmetric dimethylarginine, Intensive care, Endothelium

## Abstract

**Background:**

Optimal balance between macro- and microcirculation in critically ill patients is crucial for ensuring optimal organ perfusion. Nitric oxide (NO) is a regulator of vascular hemostasis and tone. The availability of NO is controlled by asymmetric dimethylarginine (ADMA), symmetric dimethylarginine (SDMA), and the availability of the NO substrates arginine and homoarginine. We investigated the changes in plasma concentrations of ADMA, SDMA, arginine, and homoarginine days 1–5 of intensive care unit (ICU) admission and the association between the change in concentration days 1–3 and 30-day all-cause mortality.

**Methods:**

Single-center cohort study of adult critically ill patients from the ICU at Copenhagen University Hospital – North Zealand. ADMA, SDMA, arginine, and homoarginine (NO-biomarkers) were measured on days 1–5. Initially, we determined the changes in NO-biomarkers days 1–5 with linear mixed models, and subsequently how the changes in NO-biomarkers days 1–3 were associated with 30-day all-cause mortality. Post-hoc we analyzed the association between plasma concentration at admission and 30-day all-cause mortality.

**Results:**

In total 567 out of 577 patients had plasma samples from days 1–5. Plasma concentrations of ADMA and arginine increased from days 1–5. SDMA concentrations increased from days 1–2, followed by a decrease from days 2–5. Concentrations of homoarginine did not change from days 1–3 but slightly increased from days 3–5. In total 512 patients were alive 3 days after ICU admission. Among these patients, a daily twofold increase in ADMA concentration from days 1–3 was associated with decreased mortality in multivariate analysis (HR 0.45; 95% CI 0.21–0.98; *p* = 0.046). An increase in SDMA, arginine, or homoarginine was not associated with mortality. Post-hoc we found that a twofold increase in ADMA or SDMA concentrations at admission was associated with mortality (HR 1.78; 95% CI 1.24–2.57; *p* = 0.0025, and HR 1.41; 95% CI 1.05–1.90; *p* = 0.024, respectively).

**Conclusions:**

Increasing ADMA concentrations on days 1–3 are inversely associated with mortality, however not with the same strength as high ADMA or SDMA concentrations at admission. We suggest that admission concentrations are the focus of future research on ADMA and SDMA as predictors of mortality or potential therapeutical targets in ICU patients.

**Supplementary Information:**

The online version contains supplementary material available at 10.1186/s13613-024-01362-7.

## Background

**Fig. 1:**
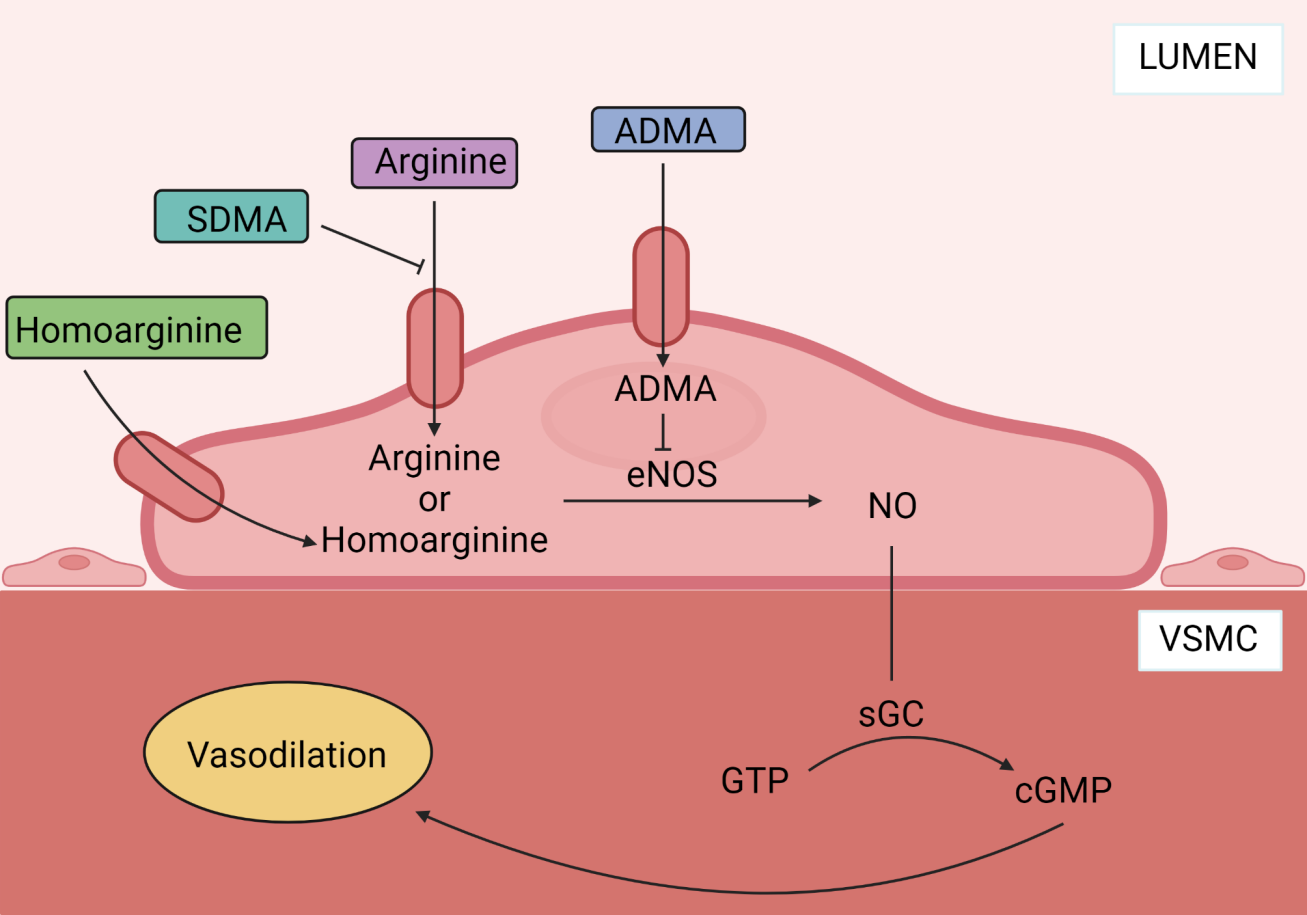
Overview of the nitric oxide (NO) metabolites. Nitric oxide (NO) is synthesized from arginine in a process catalysed by nitric oxide synthase (NOS) (in the endothelial cell endothelial NOS (eNOS)). NOS is inhibited by asymmetric dimethylarginine (ADMA). Further, the production is indirectly inhibited by symmetric dimethylarginine (SDMA), which competes with arginine for transport into the cell. Homoarginine is an alternative substrate for NOS. Within the vascular smooth muscle cell (VSMC), NO activates soluble guanylate cyclase (sGC) converting guanosine triphosphate (GTP) into cyclic guanosine monophosphate (cGMP) ultimately leading to vasodilation

Circulatory failure leading to hypoperfusion and multiple organ failure is a common problem in critically ill patients with the need for intensive care unit (ICU) treatment. Achieving the right balance between a sufficient macrocirculation, that ensures oxygen supply to all organs, and a sufficient microcirculation, that ensures the correct distribution of oxygen within each organ, is important to increase the chance of survival. Nitric oxide (NO) plays a key role in vascular homeostasis by regulating vascular tone, platelet anti-aggregation, leucocyte adhesion, and inhibition of vascular smooth muscle proliferation [1]. NO is synthesized from arginine in a process catalyzed by NO synthases (endothelial-, neuronal- and inducible NO synthase) and is upregulated in critical illness [2, 3]. The production of NO is affected by several endogenous mediators including the methylated arginines asymmetric dimethylarginine (ADMA) and symmetric dimethylarginine (SDMA), which are by-products of proteolysis. ADMA inhibits NO synthase and SDMA competes with the hCAT-2B amino acid transporter transporting arginine into the cell [4, 5] (Fig. 1). High bioavailability of NO leads to vasodilation and hypotension, whereas low bioavailability leads to impaired microcirculation [6].

High plasma concentrations of ADMA and SDMA are associated with increased mortality [7–18]. In contrast, arginine, the substrate for NO, is less investigated. Evidence from patients with out-of-hospital cardiac arrest points to an association with mortality [19], but no association has been reported from cohorts with a broader selection of ICU patients [7, 11, 20]. Homoarginine is an alternative substrate for NO synthase and an inhibitor of arginase (thereby increasing NO bioavailability) [21–23]. In a recent study of 1155 adult ICU patients, homoarginine was associated with hospital mortality [7].

From a clinical perspective, it is important to investigate whether mediators of the NO system should be measured continuously or whether single measurements are sufficient for prognostication or commencement of treatment. Changes in concentration over time of ADMA, SDMA, arginine, and homoarginine in ICU patients, have only been sparsely investigated and only with a limited number of patients and low completeness of follow-up samples [9, 10, 12, 13, 16, 17, 20, 24–27].

We aimed to describe the changes in plasma concentrations of ADMA, SDMA, arginine, and homoarginine during the first 5 days of ICU admission and to investigate the association between the change in concentration from day 1 to 3 and 30-day all-cause mortality.

## Methods

This manuscript is reported in accordance with the STROBE guidelines [28] (Additional File 1).

### Design, setting, and participants

This study is a sub-study of the Metabolomics study which is described in detail elsewhere [29]. Other studies based on the Metabolomics cohort include [29–31]. In brief, the Metabolomics study was a single-center cohort study conducted at the mixed ICU at Copenhagen University Hospital – North Zealand between November 2016 and June 2019. Inclusion criteria were acutely admission to ICU, age > 18 years, and expected admittance > 24 h. Patients were excluded if informed consent was not obtainable or active treatment was deemed futile. Initially, patient participation was approved by a trial guardian. Subsequently, informed consent was obtained from the patient’s next of kin and/or from the patient when he or she was considered capable hereof [29]. The study was approved by the local ethics committee (H-17027963) and the Danish Data Protection Agency (I-suite nr.: 04673 and 04674).

Each patient had one daily study blood sample taken for five consecutive days if the patient was admitted to the ICU. In the present study, we included all patients who had at least one blood sample available from the five-day study period. In the present study, a day 1 sample was defined as a blood sample taken within 24 h of ICU admission. A day 2 sample was defined as a sample taken on the subsequent calendar date, a minimum of eight hours after the day 1 sample. Likewise, a day 3 sample was defined as a sample taken on the subsequent date to the day 2 sample, etc.

### Measurements

EDTA plasma was separated after collection and stored at -80 °C until analysis. The concentrations of ADMA, SDMA, arginine, and homoarginine were analyzed in duplicate with enzyme-linked immunosorbent assays (ELISA) (DLD Diagnostika, Hamburg, Germany; ADMA/Arginine ELISA EA207/192; SDMA human ELISA EA214/96; Homoarginine ELISA EA205/96). Standards with a concentration of 0 were set to 0.01 to allow for logarithmic transformation and drawing of the standard curves. The standard range for each kit was: ADMA 0.2-3 µmol/L, SDMA 0.2-3 µmol/L, arginine 5-300 µmol/L and homoarginine 0.3-7 µmol/L. In 235 samples (12%) the concentration of SDMA and/or homoarginine was above the ELISA kit standard range. These samples were remeasured after a twofold or fivefold dilution. In one sample the homoarginine concentration was still over the standard range after fivefold dilution. The concentration of this sample was fixed at 35 µmol/L (5x the upper standard range of 7 µmol/L).

### Outcomes

First, we investigated the changes in concentrations over time of ADMA, SDMA, arginine, and homoarginine days 1–5. Secondly, we investigated the associations between the changes in the NO-biomarkers days 1–3 and 30-day all-cause mortality and between concentration of the NO-biomarkers at admission and 30-day all-cause mortality.

### Subgroup analyses

We investigated whether the following subgroups had different changes in NO-biomarkers days 1–5 [32]: (1) patients with septic shock vs. without septic shock (as defined by the SEPSIS-3 criteria [33]), (2) patients with septic shock and different severities of sepsis-induced endotheliopathy (divided into three groups according to the level of soluble thrombomodulin (sTM) < 4 ng/mL vs. 4–10 ng/mL vs. >10 ng/mL), (3) patients treated with dialysis (continuous renal replacement therapy (CRRT) or hemodialysis (HD)) at any day during the five day inclusion period vs. not treated with dialysis, (4) patients with acute kidney injury (AKI) vs. no AKI. AKI was defined as KDIGO [34] stage ≥ 2.

### Statistical analysis

Analyses were conducted according to a previously published statistical analysis plan available from our institution’s website [32] (Additional file 2). We used linear mixed models [35] with random intercept to investigate the changes in NO-biomarkers days 1–5. Each NO-biomarker (ADMA, SDMA, arginine, and homoarginine) was analyzed separately. We modeled time as three-knot cubic splines and the NO-biomarkers with log2. In the primary analyses, we allowed the linear mixed model to account for missing values through maximum likelihood inference.

We analyzed the associations between changes in the NO-biomarkers days 1–3 and 30-day all-cause mortality using Cox regression. We included patients who were still alive on day 3 after ICU admission. As a predictor in the Cox regression model, we used the slope of a linear model of the NO-biomarker days 1–3 for each patient. NO-biomarker concentrations were modelled with log2. The Cox regression model was adjusted for age, sex, major cardiovascular disease (history of heart failure, myocardial infarction or stroke), diabetes, hypertension, kidney failure (assessed as a four-level categorical variable with the groups: no AKI or chronic kidney injury (CKD), AKI without CKD, AKI and CKI, and CKI without AKI) and Model for End-Stage Liver Disease (MELD) score [36, 37]. The MELD score was modeled as a three-knot cubic spline to improve model fit. Calculations of the MELD score were modified so that patients who received dialysis within the first 24 h after ICU admission were assigned a fixed creatinine level of 4 mg/dL. For the primary analyses missing values of NO-biomarkers and adjusting variables used in the Cox regressions were imputed using multiple imputations. Post-hoc we decided to also conduct the Cox regression analyses with the NO-biomarker concentration at admission as the predictor variable. Since the slope and the concentration at admission were strongly correlated, we could not have them in the same regression model. To allow comparison between the post-hoc analysis of the association between admission concentrations and mortality and the primary analysis of the association between the changes in NO-biomarkers days 1–3 and mortality, we only included patients alive on day 3 in the post-hoc analysis. We also drew post-hoc Kaplan Meier curves stratified by the admission NO-biomarker concentrations in quartiles. Differences between the groups were analyzed with the log-rank test. Post-hoc univariate Cox regression analyses were performed in the subgroups septic shock, AKI, dialysis, and sTM group based upon admission variables.

To account for the potential bias of missing blood samples we performed the following pre-specified sensitivity analyses [32]: (1) Worst- and best-case assessments with a worst-case scenario where missing values of patients who died within the five-day study period were assigned the 90th percentile of ADMA and SDMA and the 10th percentile of arginine and homoarginine. Meanwhile, missing values of patients discharged from ICU within the study period were assigned the 10th percentile for ADMA and SDMA and the 90th percentile for arginine and homoarginine, (2) a best-case scenario with values assigned opposite of 1), (3) complete case analysis, and the following post-hoc sensitivity analysis: (4) last observation carried forward. Sensitivity analyses were not performed on the post-hoc analyses of NO-biomarkers at admission since the samples missing at admission were missing completely at random.

All analyses were performed with R statistical software, version 4.3.0 [38]. A p-value < 0.05 was considered statistically significant.

## Results

**Fig. 2:**
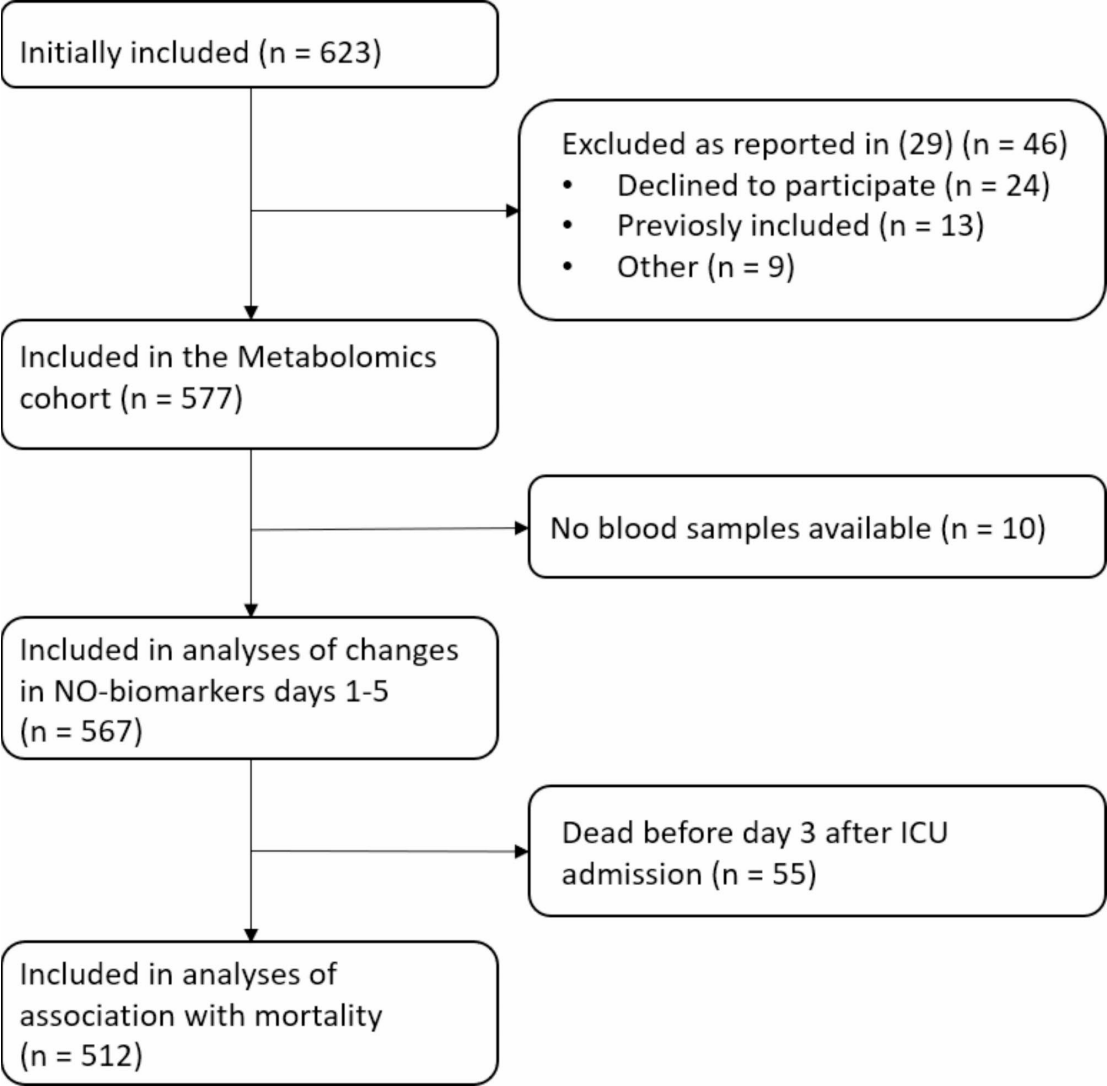
Flowchart of the inclusion process

The full Metabolomics cohort consisted of 577 patients, hereof we included 567 patients in the analyses of changes over time in NO-biomarkers days 1–5 and 512 patients, who were still alive on day 3, in the analyses of the association with 30-day all-cause mortality (Fig. 2). Of the 567 patients, 327 (58%) were male and the median age was 71 years (interquartile range (IQR) 63–79 years). All patients were critically ill with a median SAPS 3 score of 64 (IQR 56–73) and 105 (19%) had septic shock (Table 1). The 512 patients who were alive 3 days after ICU admission, tended to be younger, less comorbid, and less critically ill than patients who died before day 3 (Table 1). Median plasma concentrations of ADMA, SDMA, arginine, and homoarginine at admission are presented in Table 1.

**Table 1:** Patient characteristics

Variable	Overall, *N* = 567^1^	Survivors at day 3, *N* = 512^1^
Age	71 (63 to 79)	71 (62 to 79)
Sex		
Female	240 (42%)	216 (42%)
Male	327 (58%)	296 (58%)
History of diabetes	140 (25%)	124 (24%)
History of hypertension	301 (53%)	268 (52%)
History of myocardial infarction	63 (11%)	55 (11%)
History of stroke	59 (10%)	49 (9.6%)
History of heart failure	51 (9.0%)	44 (8.6%)
History of cronic kidney disease	88 (16%)	75 (15%)
Type of admission		
Medical	428 (75%)	390 (76%)
Surgical	139 (25%)	122 (24%)
KDIGO score ≥ 2	152 (27%)	130 (25%)
Septic Shock^2^	105 (19%)	87 (17%)
MELD score	11 (8 to 20)	11 (8 to 20)
(Missing)	8	7
SAPS 3	64 (56 to 73)	63 (55 to 72)
(Missing)	12	11
sTM, ng/mL	10 (5 to 18)	9 (5 to 17)
(Missing)	45	44
ADMA, µmol/mL	0.63 (0.49 to 0.80)	0.62 (0.49 to 0.78)
(Missing)	40	40
SDMA, µmol/mL	1.03 (0.70 to 1.67)	0.98 (0.70 to 1.59)
(Missing)	40	40
Arginine, µmol/mL	39 (28 to 59)	39 (28 to 58)
(Missing)	40	40
Homoarginine, µmol/mL	0.77 (0.48 to 1.19)	0.77 (0.50 to 1.22)
(Missing)	40	40

^1^Median (25–75%); n (%)

^2^Defined as suspected or proven infection with need for vasopressor treatment and plasma lactate ≥ 2 mmol/L

KDIGO = kidney disease - improving global outcomes

MELD = Model for End-stage Liver Disease

SAPS = simplified acute physiology score

sTM = soluble thrombomodulin

ADMA = asymmetric dimethylarginine

SDMA = symmetric dimethylarginine

**Fig. 3:**
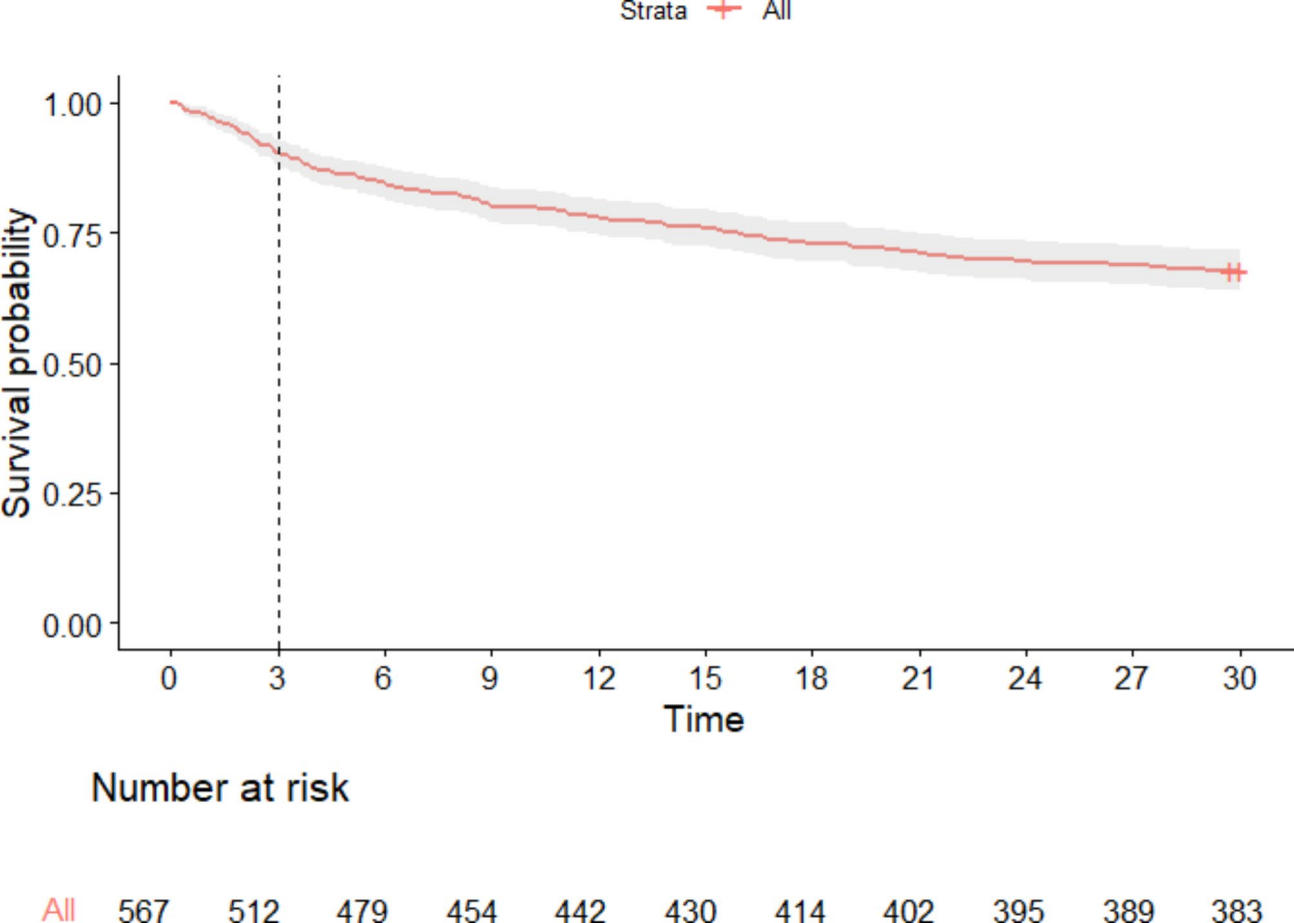
Kaplan Meier curve of the overall survival probability

From the entire cohort of 567 patients, 184 patients (32%) died within 30 days of ICU admission (Fig. 3). A total of 55 patients (9.7%) died before day 3 and were not included in the survival analyses. Of these 55 patients, 18 (33%) had septic shock at admission and 52 (95%) had multiple organ failure (defined as a Sequential Organ Failure Assessment (SOFA) score of ≥ 2 in minimum 2 of the domains respiration, circulation, central nervous system, kidney, and liver [29]). Of the 512 patients, who lived longer than day 3, 129 (25%) died before day 30, 87 (17%) had septic shock and 483 (94%) had multiple-organ failure at admission to ICU. A total of 100 (20%) of the 512 patients died while they were still in the ICU.

### Changes over time in ADMA, SDMA, arginine, and homoarginine concentrations days 1–5

ADMA and arginine concentrations consistently increased during the first 5 days of ICU admission, whereas SDMA increased from day 1 to 2 followed by a decrease from day 2 to 5. Homoarginine concentrations showed minor fluctuations (Table 2).

**Table 2:** Change in NO-biomarker concentrations during the first 5 days of ICU admission

		Change in mean concentration (95% CI)	*P* value
**ADMA**			**< 0.001**
	Day 1 to 2	11% (7–14%)	< 0.001
	Day 2 to 3	6% (4–10%)	< 0.001
	Day 3 to 4	7% (4–11%)	< 0.001
	Day 4 to 5	4% (0–9%)	0.055
**SDMA**			**< 0.001**
	Day 1 to 2	9% (5–13%)	< 0.001
	Day 2 to 3	-4% (-8-(-1)%)	0.013
	Day 3 to 4	-7% (-10-(-3)%)	< 0.001
	Day 4 to 5	-2% (-7-2%)	0.3
**Arginine**			**< 0.001**
	Day 1 to 2	18% (13–24%)	< 0.001
	Day 2 to 3	21% (15–27%)	< 0.001
	Day 3 to 4	16% (10–24%)	< 0.001
	Day 4 to 5	12% (4–20%)	0.001
**Homoarginine**			**0.012**
	Day 1 to 2	0% (-4-4%)	> 0.9
	Day 2 to 3	0% (-4-4%)	> 0.9
	Day 3 to 4	4% (-1-9%)	0.093
	Day 4 to 5	4 (-1-10%)	0.2

Change in mean concentration from day to day during the first 5 days of ICU admission was calculated with linear mixed models

NO = nitric oxide, CI = confidence interval, ADMA = asymmetric dimethylarginine, SDMA = symmetric dimethylarginine

**Fig. 4:**
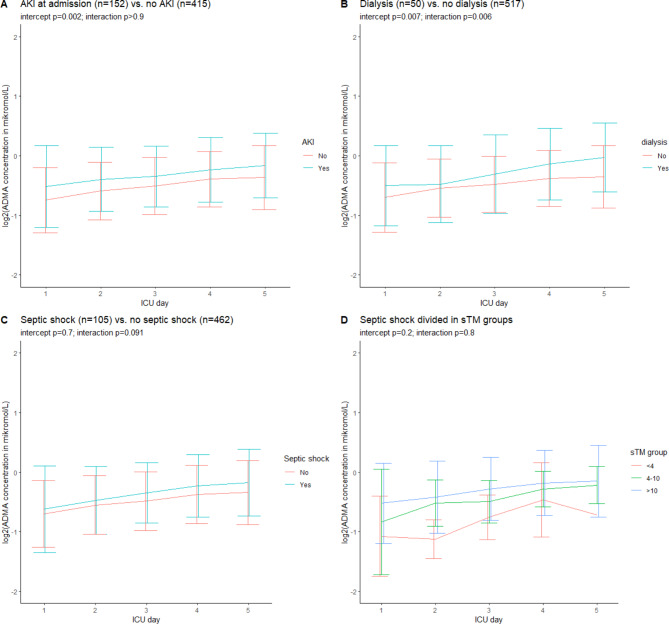
Changes in ADMA concentration days 1–5 in subgroups. Figures show mean +/- sd. AKI is defined as a KDIGO [34] score ≥ 2 at admission. Dialysis is defined as treatment with continuous renal replacement therapy (CRRT) or hemodialysis (HD) on any day during the 5-day study period. Septic shock is defined as a suspected or proven infection with a need for vasopressor treatment and plasma lactate ≥ 2 mmol/L at admission. sTM groups are defined as sTM < 4 ng/mL (*n* = 5) versus 4–10 ng/mL (*n* = 21) versus > 10 ng/mL (*n* = 77). ADMA = asymmetric dimethylarginine, sTM = soluble thrombomodulin. Corresponding figures for the other NO-biomarkers are presented in Additional File 3

Patients with AKI at admission (*n* = 152) had significantly higher ADMA and SDMA plasma concentrations and significantly different time courses of SDMA and homoarginine (Fig. 4A and Additional File 3 Supplemental Fig. 2A and 4A and Supplemental Tables 1, 5 and 13). Patients treated with dialysis on any day during the five-day study period (*n* = 50) had significantly higher ADMA and SDMA plasma concentrations and significantly different time courses of ADMA, arginine, and homoarginine (Fig. 4B and Additional File 3 Supplemental Fig. 2B, 3B and 4B and Supplemental Tables 2, 6, 10 and 14). Patients with septic shock (*n* = 105) had similar changes in concentrations of ADMA and SDMA as patients without septic shock (Fig. 4C and Additional File 3 Supplemental Fig. 2C and Supplemental Tables 3 and 7). In contrast, changes in concentrations of arginine and homoarginine were significantly different, and arginine concentrations were significantly higher in patients without septic shock (Additional File 3 Supplemental Fig. 3C and 4C Supplemental Tables 11 and 15). When the patients with septic shock were further divided by the admission level of sTM the only significant difference was the SDMA plasma concentration, which increased with higher sTM level (Additional File 3 Supplemental Fig. 2D and Supplemental Table 8).

### Changes in ADMA, SDMA, arginine, and homoarginine days 1–3 and 30-day all-cause mortality

A daily twofold increase in ADMA concentration from days 1–3 was associated with decreasing 30-day mortality (HR 0.45; 95% CI 0.21–0.98; *p* = 0.046) (Table 3 and Additional File 4). An increase in SDMA, arginine, or homoarginine was not associated with mortality (Table 3). At admission a twofold higher concentration of ADMA and SDMA was associated with increased mortality (HR 1.78; 95% CI 1.24–2.57; *p* = 0.0025, and HR 1.41; 95% CI 1.05–1.90; *p* = 0.024, respectively) (Table 3). Arginine and homoarginine concentrations at admission were not associated with mortality (Table 3).

**Table 3:** NO-biomarkers and 30-day all-cause mortality – overview of multivariate Cox regressions

	Adjusted HR (95% CI)^#^	*P* value
ADMA change day 1–3*^$^	0.45 (0.21–0.98)	0.046
SDMA change day 1–3*^$^	1.07 (0.53–2.17)	0.84
Arginine change day 1–3*^$^	0.70 (0.44–1.12)	0.14
Homoarginine change day 1–3*^$^	0.84 (0.47–1.50)	0.55
ADMA admission^$^	1.78 (1.24–2.57)	0.0025
SDMA admission^$^	1.41 (1.05–1.90)	0.024
Arginine admission^$^	1.19 (0.94–1.51)	0.16
Homoarginine admission^$^	1.15 (0.97–1.38)	0.12

^#^Adjusted for age, sex, major cardiovascular disease, diabetes, hypertension, kidney failure, and model for end-stage liver disease (MELD) score

*The change from days 1–3 was estimated as a slope from a linear model for each patient

^$^All NO-biomarkers (change days 1–3 and admission) were transformed with log2 and analyzed separately. See Additional File 4 for complete regression outputs

NO = nitric oxide, HR = hazard ratio, CI = confidence interval, ADMA = asymmetric dimethylarginine, SDMA = symmetric dimethylarginine

**Fig. 5:**
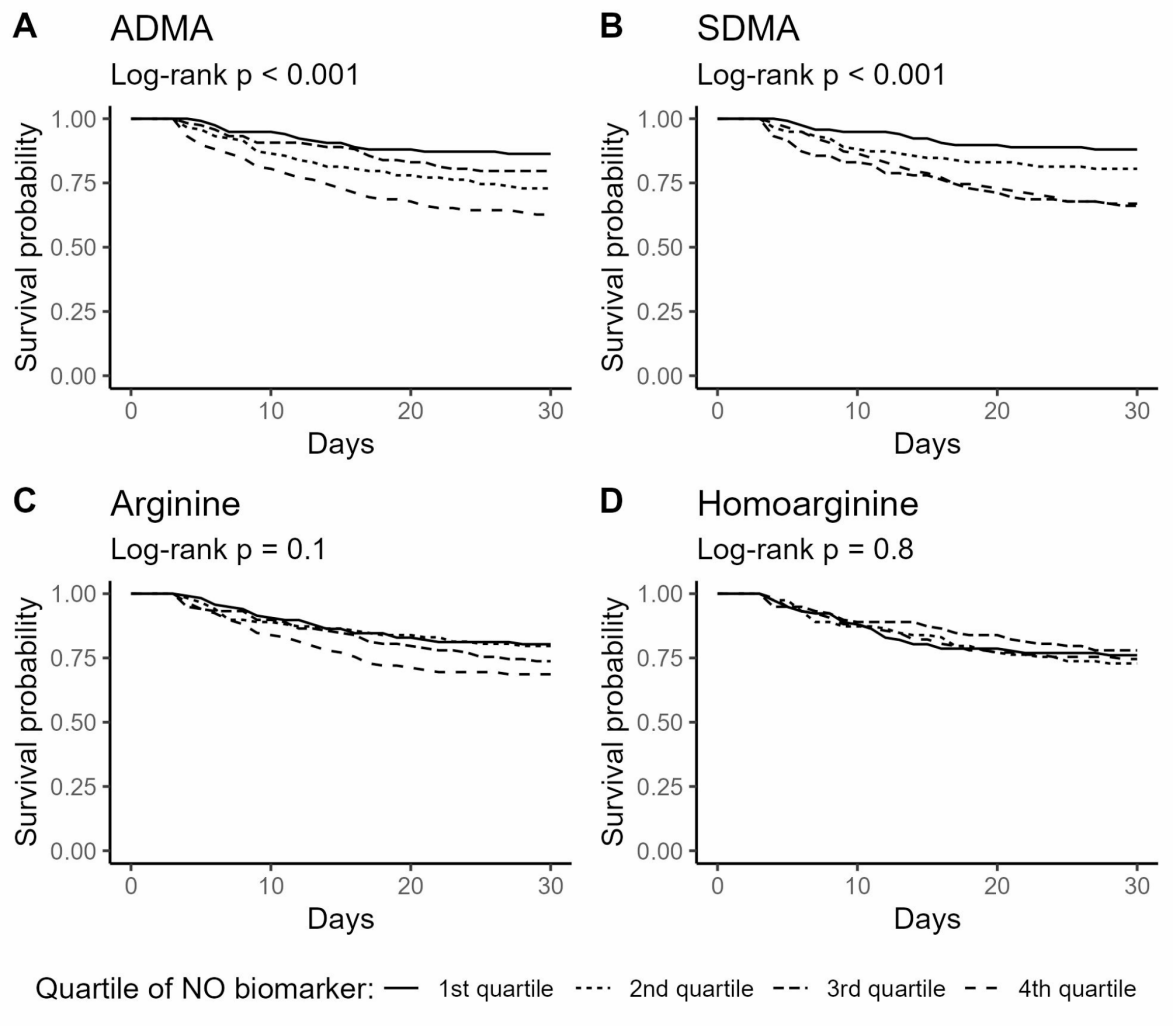
Survival curves stratified by NO-biomarker admission concentration in quartiles. NO = nitric oxide, ADMA = asymmetric dimethylarginine, SDMA = symmetric dimethylarginine

In conformity with the results of the Cox regressions patients with an admission ADMA concentration in the 4th quartile, and patients with an admission SDMA concentration in the 3rd or 4th quartile had significantly different survival curves compared to the remaining patients (Fig. 5). Neither the presence of septic shock, AKI, dialysis nor severe endotheliopathy at admission affected the associations between NO biomarkers and mortality (Additional File 5 Supplemental Figs. 5–12).

### Sensitivity analyses

An overview of the missing samples with reasons is given in Additional File 6 (Supplemental Fig. 13). Sensitivity analyses show good agreement with the primary analyses from days 1–3, with more uncertain results hereafter (Additional File 6 Supplemental Fig. 14A-D and Supplemental Tables 27–41).

## Discussion

The aim of this study was twofold: firstly, to describe the changes in plasma concentrations of ADMA, SDMA, arginine, and homoarginine during the first 5 days of ICU admission and secondly, to investigate the association between the change in concentration from day 1 to 3 and 30-day all-cause mortality.

We found that all four NO-biomarkers changed during the first 5 days of ICU admission, with increasing concentrations of ADMA and arginine, and smaller fluctuations in concentrations of SDMA and homoarginine. In subgroup analyses, most in-between group difference was seen in the kidney failure groups (AKI and dialysis subgroups). This is not surprising since it is well known that plasma concentrations of ADMA and SDMA are elevated in patients with renal impairment [39]. SDMA is highly correlated with glomerular filtration rate suggesting that SDMA could be a new marker for renal function [40]. We did not find a difference in plasma concentrations of ADMA or SDMA in patients with septic shock compared to patients without septic shock. Several cohort studies have shown that ADMA concentrations in septic patients are elevated compared to healthy controls [20, 24–26] and that increased ADMA plasma concentrations are associated with increased SOFA scores [17, 24]. However, in line with our results, ADMA concentrations did not differ in critically ill patients with and without sepsis in a cohort of 255 patients neither at admission nor on day 7 [9].

The increasing ADMA concentration in the overall ICU population agrees with results from previous smaller cohorts [9, 12, 20, 24, 26], however, not consistent in all cohorts [13, 25]. The reason for increasing ADMA plasma concentration is unknown and different theories have been suggested. Approximately 90% of ADMA is metabolized by the enzyme dimethylarginine dimethylaminohydrolase (DDAH) [4] and it has been suggested that DDAH is impaired by oxidative stress in endothelial dysfunction thus leading to the accumulation of ADMA [1]. Other possible explanations are that increasing ADMA concentration is a sign of recovery as NO inhibits DDAH activity and thereby indirectly increases ADMA concentration [12] or that increasing ADMA is a result of worsening liver function as the liver has been shown to play an important role in metabolizing ADMA [12, 41].

Changes in plasma concentrations of SDMA, arginine, and homoarginine are even less investigated. A significant daily increase of SDMA for 12 days was observed in 72 patients with severe sepsis [13]. Other cohorts reported non-significant changes in SDMA concentrations [10, 12, 20, 26]. Increasing arginine concentrations, similar to our results, have been reported in other cohorts of ICU patients [12, 20, 26], though not consistently in all cohorts [25]. Repeated measurements of homoarginine in ICU patients have, to our knowledge, only been investigated by one study, which reported a significant decrease in concentration [20]. In summary, existing evidence (including the results of the present study) points to increasing plasma concentrations of ADMA and arginine during the initial days of ICU stay combined with more stable concentrations of SDMA and homoarginine. Whether the increasing plasma concentrations result in decreased NO bioavailability depends on the magnitudes of the increase of ADMA and arginine.

Regarding the association with mortality, we found that increasing ADMA concentration over the first 3 days of ICU admission was inversely associated with 30-day all-cause mortality. This finding contrasts with a study of 79 surgical ICU patients showing that increasing ADMA concentration from day 0 to 2 was associated with increased mortality [42]. One of the great challenges, when investigating changes over time of a biomarker in ICU patients, is how to account for the potential bias of patients who die and patients who are discharged from ICU within the study period. After thorough statistical discussions, we decided to only include patients alive 3 days after ICU admission and to estimate the change from day 1 to 3 from the slope of a linear model based on the daily samples from each patient. We were fully aware that this method of analysis would ignore some of the in-between patient variation, but a close correlation between the change in concentrations from days 1–3 (hence the slope of the linear model) and the admission concentration might also cause an analytical problem. The correlation is graphically presented in Supplemental Fig. 15 (Additional File 7) and shows that a great majority of patients with an admission concentration in the 1st quartile have increasing ADMA concentrations on days 1–3 (positive slope), whereas patients in the 4th quartile both present with increasing and decreasing concentrations days 1–3 (positive and negative slopes). Additionally, there is a risk of survivorship bias since we excluded patients who died before day 3 since we cannot evaluate the association between time course and mortality for patients who die within the study period. The results of the sensitivity analyses show that our primary analyses best align with the results of the complete case and last observation carried forward sensitivity analyses and thereby outline the difficulty of dealing with the potential bias of missing blood samples due to death or discharge from ICU.

Post-hoc we decided to investigate the association between plasma concentrations at admission and 30-day all-cause mortality and found high admission concentrations of ADMA and SDMA to be strongly associated with increased mortality in multivariate analyses. As outlined in the introduction section of this paper, these findings agree with results from previous studies [7–18]. Even though subgroup analyses of the changes over time showed that patients with AKI had higher plasma concentrations of ADMA and SDMA, subgroup analyses of the Cox regressions showed no differences in mortality. Likewise, kidney failure did not affect the associations between NO-biomarker concentrations and mortality in our multivariate statistical analyses (data shown in Additional File 4). We found this result surprising since increased plasma concentrations of ADMA and SDMA are associated with increased creatinine [15, 17]. However, at least in our study, this association was not strong enough to affect mortality. Likewise, we expected septic shock to affect the association between NO-biomarker concentrations and mortality. Septic shock is a clinical diagnosis covering a heterogeneous patient population with different underlying pathologies. Alterations in the NO system are likely more important in some pathologies than others which may blur the effect in this wide cohort of ICU patients.

This study has strengths and limitations of important consideration. Strengths include that the cohort is one of the largest in this field. Further, we published a statistical analysis plan, had a complete follow-up regarding the outcome, and performed extensive sensitivity analyses of the potential bias of missing samples, not only presenting a raw linear mixed model or complete case analysis as previous studies including repeated measurements in this field (linear mixed model: [12, 13, 27], complete case: [9, 10, 17, 20, 24–26]). However, there are also limitations. First, this is a single-center study, which reduces generalizability. Second, no screening log was kept, and therefore we cannot account for potential differences between patients included in the study and patients not included. Third, as already stated there are fundamental statistical challenges when analyzing the association between repeated measurements of a biomarker and mortality in a population with a high (and early) mortality rate. Fourth, this is an observational cohort study with an explorative design, so all results should be evaluated with this in mind.

Based on our results, and with the strengths and limitations in mind, we postulate that the admission concentrations of ADMA and SDMA have a greater impact on mortality than the change during the first days of ICU stay. This conforms with a cohort study in 255 medical ICU patients, which reported an association between high ADMA concentration at admission and both increased ICU- and 3-year mortality but also demonstrated that the addition of the individual increase in ADMA between admission to ICU and day 7 did not improve the prognostic value of ADMA [9].

There are different ways increased knowledge about the nitric oxide system can improve future intensive care. Plasma ADMA and SDMA have both been suggested as a method of risk stratification in septic patients [17] and ADMA as a new potential target when treating ICU patients with circulatory impairment [6]. At first, the idea of ADMA and SDMA for risk stratification might seem challenged by our study since we found that increasing ADMA values over time are associated with lower mortality and did not find significantly different concentrations between patients with and without septic shock. However, in our opinion, the interpretation of the present study must be that the predictive signal is in high concentrations of ADMA and SDMA at admission and not in changes over time or in specific between-group differences. This statement is supported by the results we present in Table 3 and Fig. 5 showing that high plasma concentrations of ADMA and SDMA at admission are strongly associated with increased mortality. The strong association between admission concentrations and mortality is a confirmation of our previous study in a cohort of 267 patients with severe sepsis and septic shock [18]. The research group behind the hypothesis of beneficial lowering of ADMA argues that lowering of ADMA has the potential to restore proper microcirculation by normalizing the intravascular NO concentration and to reduce inflammation, the production of reactive oxygen species, and most importantly reduce mortality [6]. Two studies have recently demonstrated that lowering of ADMA is possible by recombinant DDAH protein and has beneficial effects on endothelial functions in vitro and in rodents [43, 44]. However, we do not regard lowering of ADMA as a future widely distributed treatment intervention, but rather as a specific intervention in patients with measured high ADMA as a targeted intervention in this subpopulation of critically ill patients.

## Conclusions

Plasma concentrations of ADMA and arginine increase during the first 5 days of ICU admission in a broad population of ICU patients while plasma concentrations of SDMA and homoarginine exert minor fluctuations. Increasing plasma concentrations of ADMA during the first 3 days of ICU admission is associated with decreased 30-day all-cause mortality, however not as strongly as the association between high plasma concentrations of ADMA and SDMA at admission and increased 30-day all-cause mortality. ADMA is a potential new diagnostic and therapeutic target in ICU patients with circulatory impairment. We suggest focusing future research on high admission concentration as the treatment trigger rather than changes in concentration during the first days of ICU admission.

### Electronic supplementary material

Below is the link to the electronic supplementary material.

Supplementary Material 1

Supplementary Material 2

Supplementary Material 3

Supplementary Material 4

Supplementary Material 5

Supplementary Material 6

Supplementary Material 7

## Data Availability

The dataset analyzed during the current study are available from the corresponding author upon reasonable request.

## Abbreviations

ICU: Intensive Care Unit
NO: Nitric oxide
ADMA: Asymmetric dimethylarginine
SDMA: Symmetric dimethylarginine
ELISA: Enzyme-linked immunosorbent assay
sTM: Soluble thrombomodulin
CRRT: Continuous renal replacement therapy
HD: Hemodialysis
AKI: Acute kidney injury
CKD: Chronic kidney injury
MELD: Model for End-Stage Liver Disease
KDIGO: Kidney disease – improving global outcomes
SAPS: Simplified acute physiology score
HR: Hazard ratio
CI: Confidence interval
IQR: Interquartile range
DDAH: Dimethylarginine dimethylaminohydrolase
SOFA: Sequential organ failure assessment score
