# Supplementary material for: Changes in nitric oxide inhibitors and mortality in critically ill patients: a cohort study
Source: Ann Intensive Care. 2024 Aug 27;14:133. doi: 10.1186/s13613-024-01362-7 (PMC11349968; doi:10.1186/s13613-024-01362-7)
Supplement: Supplementary file 2 — Supplementary Material 2 [file 13613_2024_1362_MOESM2_ESM.pdf]

Date: January 24th 2023

Statistical analysis plan for "Alterations in the nitric oxide system during the first 5 days of ICU admission"

Karoline Myglegård Mortensen[1], Theis Skovsgaard Itenov[2], Jakob Stensballe[3,4], Morten Heiberg Bestle[1,5]

[1]Department of Anesthesiology and Intensive Care, Copenhagen University Hospital – North Zealand, Copenhagen, Denmark, [2]Department of Anesthesiology and Intensive Care, Bispebjerg and Frederiksberg Hospitals, Denmark, [3]Section for Transfusion Medicine, Capital Region Blood Bank, Rigshospitalet, Copenhagen, Denmark, [4]Department of Anesthesiology, Surgery and Trauma Center, Centre of Head and Orthopedics, Copenhagen University Hospital – Rigshospitalet, Denmark, [5]Department of Clinical Medicine, University of Copenhagen, Copenhagen, Denmark

Background

The nitric oxide (NO) system play an important role in the pathophysiology of circulatory instability in critically ill patients[1]. Since NO is a volatile gas, it is difficult to measure its concentration in plasma directly, however it is possible to measure the concentration of the NO substrates l-arginine and homoarginine, the endogen NO synthase inhibitor asymmetric dimethylarginine (ADMA) and symmetric dimethylargine (SDMA) inhibiting l-arginine uptake[2]. All four NO biomarkers have been shown to be associated with mortality in critically ill patients[3–8]. Current knowledge is primarily based on baseline blood samples taken at the diagnosis of septic shock or admission to ICU. To assess the clinical relevance of the NO biomarkers we need to know how their concentrations change over time.

Hypothesis

It is our hypothesis that a reduced bioavailability of NO is associated with increased mortality. Reduced bioavailability of NO can be caused be a combination of high plasma concentrations of ADMA and SDMA and low plasma concentrations of l-arginine and homoarginine. Over time we hypothesize that a further increase in ADMA and/or SDMA concentration will be associated with increased mortality. Likewise, a decrease in l-arginine and/or homoarginine are hypothesized to be associated with increased mortality.

Study population

567 mixed ICU patients with blood samples in the biobank of the Metabolomics study. The blood samples were collected at the Intensive Care Unit (ICU) at Copenhagen University Hospital – North Zealand from November 2016 to June 2019. The Metabolomics cohort are described in details elsewhere[9].

Analysis 1: Changes in nitric oxide biomarkers during the first five days of ICU stay

Aim: Determine the changes in concentrations of the NO biomarkers l-arginine, homoarginine, ADMA and SDMA during the first 5 days of ICU stay

Patients: All in cohort

Outcome: Description of the development over time in concentration of ADMA, SDMA, l-arginine and homoarginine respectively.

Subgroup analyses:

- Patients with septic shock vs. without septic shock (as defined by the SEPSIS-3 criteria)
- Patients with septic shock and level of soluble thrombomodulin (sTM) < 4 ng/ml vs. sTM 4-10 ng/ml vs. > 10 ng/ml.
- Patients treated with dialysis (continuous renal replacement therapy (CRRT) or haemodialysis (HD)) at any day during the 5-day inclusion period vs. not treated with dialysis.
- Patients with acute kidney injury (AKI) defined by KDIGO[10] stage ≥ 2 vs. no AKI.

Statistical analysis:

We will use linear mixed models[11] to describe the alterations of the nitric oxide biomarkers over time. The 4 biomarkers (ADMA, SDMA, l-arginine and homoarginine) will be analyzed separately. We will model time as a fixed effect. If the effect of time is not linear, we will model time with splines, polynomial, or logarithm as appropriate. We will allow for random intercept for each patient and random slopes for the effect of time. We will check the assumptions of the model trough inspection of normality of residuals.

As this is a cohort with critically ill patients in the need of intensive care not all included patients survive through the whole study period (5 days). Likewise, there is a subpopulation of included patients not ill enough to stay admitted to the ICU through the whole study period. Both scenarios contain problems with handling missing data because they are not missing at random (MAR). To account for the potential bias of the above we will perform the following sensitivity analyses:

- "Worst case" scenario: patients who die within the study period will be assigned a "worst case" value of the biomarker after they die and patients who get discharged will be assigned a "best case" value of the biomarker after the time of discharge. As the "worst case" value we will use the 90th percentile for SDMA and ADMA, and the 10th percentile for l-arginine and homoarginine. As the "best case" value we will use the 10th percentile for SDMA and ADMA, and the 90th percentile for l-arginine and homoarginine.
- "Best case" scenario: values assigned opposite of described in the "worst case" scenario
- Complete case analysis

The three models from the sensitivity analyses will be compared to the primary model. In the primary model we will allow for the mixed model to imputate the missing values through likelihood inference (assuming MAR). The subgroup analyses defined above will be based on the primary model.

Analysis 2: Effect of changes in nitric oxide biomarkers during the first 3 days of ICU stay on 30-day all-cause mortality

Aim: Determine whether changes in different directions over time are associated with mortality.

Patients: Alive on day 3

Exposure / risk factor: Time trends in NO biomarkers day 1-3

Outcomes: 30-day all-cause mortality.

Statistical analysis:

To evaluate the effect of biomarker concentration over time on mortality we will use the estimates of intercept (baseline value) and slope from the linear mixed model for each patient as covariates in a cox regression model. In the cox regression model, we will allow for an interaction between the estimates for intercept and slope, if these are not strongly correlated (r-squared < 0.5). If they are strongly correlated, we will use the slope. We will adjust the cox regression model for the following baseline covariates:

- Age
- Gender
- Previous major cardiovascular disease defined as a diagnosis of heart failure, myocardial infarction or stroke
- Diabetes
- Hypertension
- Kidney failure defined as a 4-level categorical variable of acute kidney injury (AKI) and/or chronic kidney disease (CKD): "no AKI or CKD", "AKI without CKD", "CKD and AKI", "CKD without AKI"
- Liver failure assed by the Model for End-Stage Liver Disease (MELD)[13,14] score

In case of missingness in the baseline covariates we will imputate these using multiple imputation. We will present the results for both the univariate and the multivariate analysis. We will check the assumptions of the cox regression model using cumulative martingale residuals. We will perform the same sensitivity analyses in part 2 as described in part 1.

All analyses will be performed using R statistical software[15]. A p-value < 0,05 will be considered statistically significant. We will not adjust for multiple comparisons as all findings will be considered exploratory.

References

1. Lambden, S., Creagh-Brown, B. C., Hunt, J., Summers, C. & Forni, L. G. Definitions and pathophysiology of vasoplegic shock. *Crit. Care* **22**, 174 (2018).
2. Blackwell, S. The biochemistry, measurement and current clinical significance of asymmetric dimethylarginine. *Ann. Clin. Biochem.* **47**, 17–28 (2010).
3. Koch, A. *et al.* Elevated asymmetric dimethylarginine levels predict short- and long-term mortality risk in critically ill patients. *J. Crit. Care* **28**, 947–53 (2013).
4. Brenner, T. *et al.* L-arginine and asymmetric dimethylarginine are early predictors for survival in septic patients with acute liver failure. *Mediators Inflamm.* **2012**, 210454 (2012).
5. Iapichino, G. *et al.* Time course of endogenous nitric oxide inhibitors in severe sepsis in humans. *Minerva Anestesiol.* **76**, 325–333 (2010).
6. Mortensen, K. M. *et al.* High Levels of Methylarginines Were Associated With Increased Mortality in Patients With Severe Sepsis. *Shock* **46**, 365–372 (2016).
7. Koch, A. *et al.* Regulation and prognostic relevance of symmetric dimethylarginine serum concentrations in critical illness and sepsis. *Mediators Inflamm.* **2013**, 413826 (2013).
8. Lee, T. F. *et al.* ADMA and homoarginine independently predict mortality in critically ill patients. *Nitric oxide Biol. Chem.* **122**–**123**, 47–53 (2022).
9. Schønemann-Lund, M. *et al.* Endotheliopathy is associated with slower liberation from mechanical ventilation: a cohort study. *Crit. Care* **26**, 1–15 (2022).
10. Kellum, J. a *et al.* KDIGO Clinical Practice Guideline for Acute Kidney Injury. *Kidney Int. Suppl.* **2**, 1–138 (2012).
11. Winter, B. Linear models and linear mixed effects models in R with linguistic applications. arXiv:1308.5499. http://arxiv.org/pdf/1308.5499.pdf (2013).
12. Bates, D., Maechler, M., Bolker, B. & Walker, S. Fitting Linear Mixed-Effects Models Using lme4. *J. Stat. Softw.* **67**, 1–48 (2015).
13. Kamath, P. S. & Kim, W. R. The Model for End-stage Liver Disease (MELD). *Hepatology* **45**, 797–805 (2007).
14. Liver and Intestinal Organ Transplanation Committee - OPTN/UNOS. Clerical changes for implementation of adding serum sodium to the MELD Score. 4–5 (2015).
15. R Core Team. R: A language and environment for statistical computing. (2019).
