# Supplementary material for: Changes in nitric oxide inhibitors and mortality in critically ill patients: a cohort study
Source: Ann Intensive Care. 2024 Aug 27;14:133. doi: 10.1186/s13613-024-01362-7 (PMC11349968; doi:10.1186/s13613-024-01362-7)
Supplement: Supplementary file 3 — Supplementary Material 3 [file 13613_2024_1362_MOESM3_ESM.docx]

**Additional File 3:** Results of linear mixed models in subgroups

1. ADMA

**Supplemental Figure 1 (= Figure 4 in the main manuscript):** Changes in ADMA concentration days 1-5 in subgroups

**
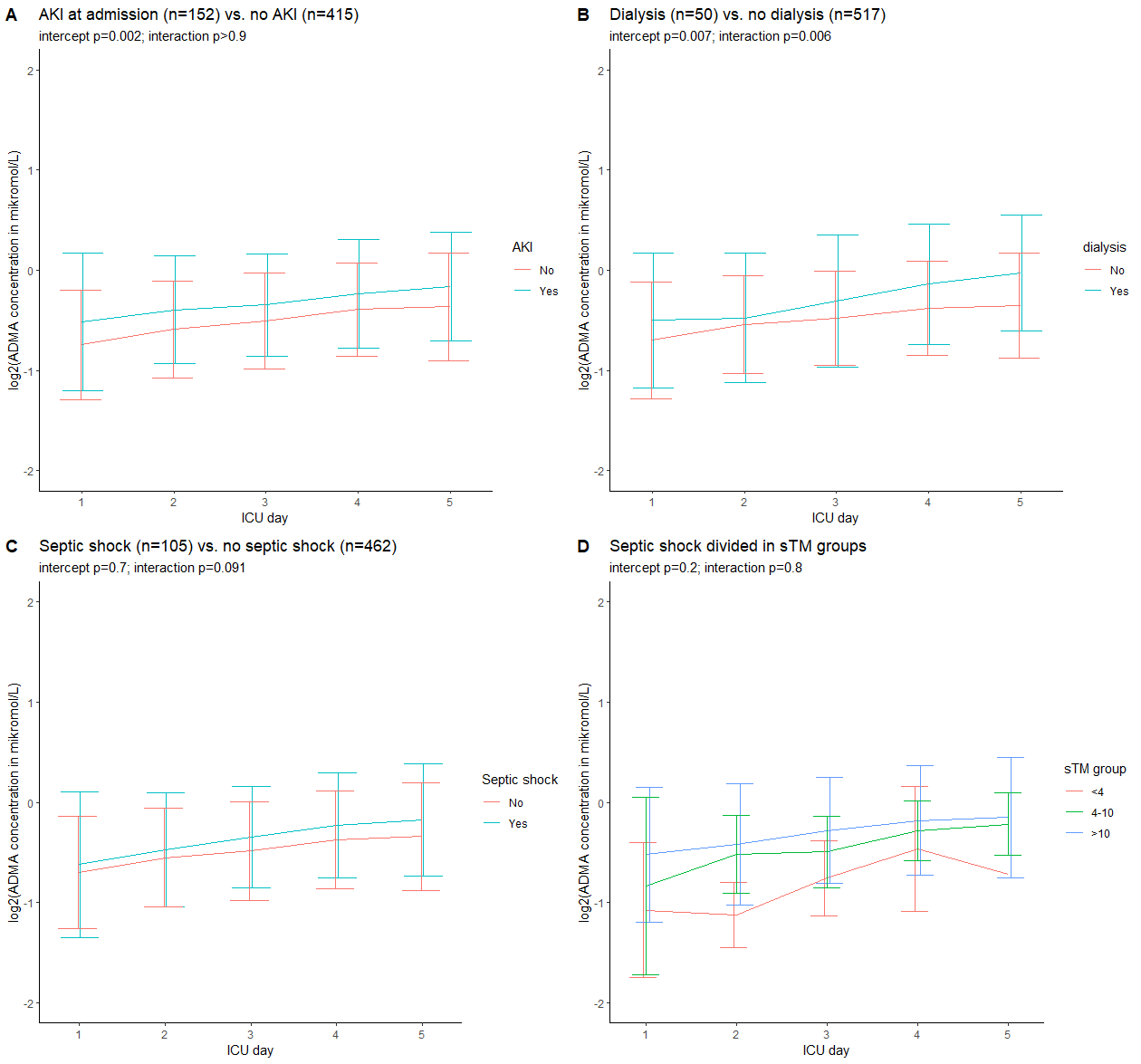
**

Figures show mean +/- sd. AKI is defined as a KDIGO(34) score ≥2 at admission. Dialysis is defined as treatment with continuous renal replacement therapy (CRRT) or hemodialysis (HD) on any day during the 5-day study period. Septic shock is defined as a suspected or proven infection with a need for vasopressor treatment and plasma lactate ≥ 2 mmol/L at admission. sTM groups are defined as sTM <4 ng/mL (n=5) versus 4-10 ng/mL (n=21) versus >10 ng/mL (n=77). ADMA = asymmetric dimethylarginine, sTM = soluble thrombomodulin.

**Supplemental Table 1:** ADMA concentrations in patients with AKI at admission (n=152) versus patients without AKI (n=415)

| **Variable** | **estimate** | **95% CI**^1^ | **p-value** |
| --- | --- | --- | --- |
| Sample time |  |  | <0.001 |
| ICU day 1-2 | 0.16 | 0.11, 0.21 |  |
| ICU day 2-3 | 0.09 | 0.03, 0.14 |  |
| ICU day 3-4 | 0.10 | 0.04, 0.16 |  |
| ICU day 4-5 | 0.06 | -0.01, 0.14 |  |
| AKI^2^ | 0.27 | 0.10, 0.43 | 0.002 |
| Sample time * AKI |  |  | >0.9 |
| ICU day 1-2 * AKI | -0.05 | -0.14, 0.05 |  |
| ICU day 2-3 * AKI | 0.01 | -0.09, 0.11 |  |
| ICU day 3-4 * AKI | 0.01 | -0.10, 0.12 |  |
| ICU day 4-5 * AKI | -0.02 | -0.15, 0.11 |  |
| ^1^CI = Confidence Interval | | | |
| ^2^KDIGO score ≥2 at admission | | | |

**Supplemental Table 2:** ADMA concentrations in patients treated with dialysis at any day during the five day study period (n=50) versus patients not treated with dialysis (n=517)

| **Variable** | **estimate** | **95% CI**^1^ | **p-value** |
| --- | --- | --- | --- |
| Sample time |  |  | <0.001 |
| ICU day 1-2 | 0.16 | 0.12, 0.20 |  |
| ICU day 2-3 | 0.08 | 0.03, 0.13 |  |
| ICU day 3-4 | 0.08 | 0.03, 0.13 |  |
| ICU day 4-5 | 0.05 | -0.02, 0.11 |  |
| Dialysis^2^ | 0.36 | 0.10, 0.62 | 0.007 |
| Sample time * Dialysis |  |  | 0.006 |
| ICU day 1-2 * Dialysis | -0.15 | -0.30, -0.01 |  |
| ICU day 2-3 * Dialysis | 0.11 | -0.03, 0.25 |  |
| ICU day 3-4 * Dialysis | 0.12 | -0.03, 0.27 |  |
| ICU day 4-5 * Dialysis | 0.03 | -0.13, 0.20 |  |
| ^1^CI = Confidence Interval | | | |
| ^2^at any day during the first five days of ICU stay | | | |

**Supplemental Table 3:** ADMA concentrations in patients with septic shock (n=105) versus patients without septic shock (n=462)

| **Variable** | **estimate** | **95% CI**^1^ | **p-value** |
| --- | --- | --- | --- |
| Sample time |  |  | <0.001 |
| ICU day 1-2 | 0.14 | 0.10, 0.19 |  |
| ICU day 2-3 | 0.08 | 0.03, 0.13 |  |
| ICU day 3-4 | 0.09 | 0.03, 0.15 |  |
| ICU day 4-5 | 0.06 | -0.01, 0.12 |  |
| Septic Shock^2^ | 0.04 | -0.15, 0.23 | 0.7 |
| Sample time * Septic Shock |  |  | 0.091 |
| ICU day 1-2 * Septic Shock | 0.02 | -0.09, 0.13 |  |
| ICU day 2-3 * Septic Shock | 0.08 | -0.03, 0.19 |  |
| ICU day 3-4 * Septic Shock | 0.03 | -0.09, 0.16 |  |
| ICU day 4-5 * Septic Shock | 0.01 | -0.14, 0.15 |  |
| ^1^CI = Confidence Interval | | | |
| ^2^Defined as suspected or proven infection with need for vasopressor treatment and plasma lactate ≥ 2 mmol/L | | | |

**Supplemental Table 4:** ADMA concentrations in patients with septic shock and admission plasma sTM concentration of <4 (n=5) versus 4-10 (n=21) versus >10 ng/mL (n=77)

| **Variable** | **estimate** | **95% CI**^1^ | **p-value** |
| --- | --- | --- | --- |
| Sample time |  |  | 0.005 |
| ICU day 1-2 | 0.11 | -0.36, 0.59 |  |
| ICU day 2-3 | 0.37 | -0.11, 0.85 |  |
| ICU day 3-4 | 0.29 | -0.19, 0.77 |  |
| ICU day 4-5 | 0.11 | -0.70, 0.93 |  |
| sTM group^2^ |  |  | 0.2 |
| <4 | — | — |  |
| 4-10 | 0.13 | -0.81, 1.1 |  |
| >10 | 0.50 | -0.38, 1.4 |  |
| Sample time * sTM group |  |  | 0.8 |
| ICU day 1-2 * sTM group 4-10 | 0.12 | -0.40, 0.65 |  |
| ICU day 2-3 * sTM group 4-10 | -0.33 | -0.87, 0.21 |  |
| ICU day 3-4 * sTM group 4-10 | -0.14 | -0.69, 0.42 |  |
| ICU day 4-5 * sTM group 4-10 | 0.03 | -0.85, 0.91 |  |
| ICU day 1-2 * sTM group >10 | 0.03 | -0.46, 0.52 |  |
| ICU day 2-3 * sTM group >10 | -0.19 | -0.69, 0.31 |  |
| ICU day 3-4 * sTM group >10 | -0.18 | -0.68, 0.32 |  |
| ICU day 4-5 * sTM group >10 | -0.07 | -0.89, 0.76 |  |
| ^1^CI = Confidence Interval | | | |
| ^2^sTM = soluble thrombomodulin (ng/mL) | | | |

1. **SDMA**

**Supplemental Figure 2:** Changes in SDMA concentration days 1-5 in subgroups

**
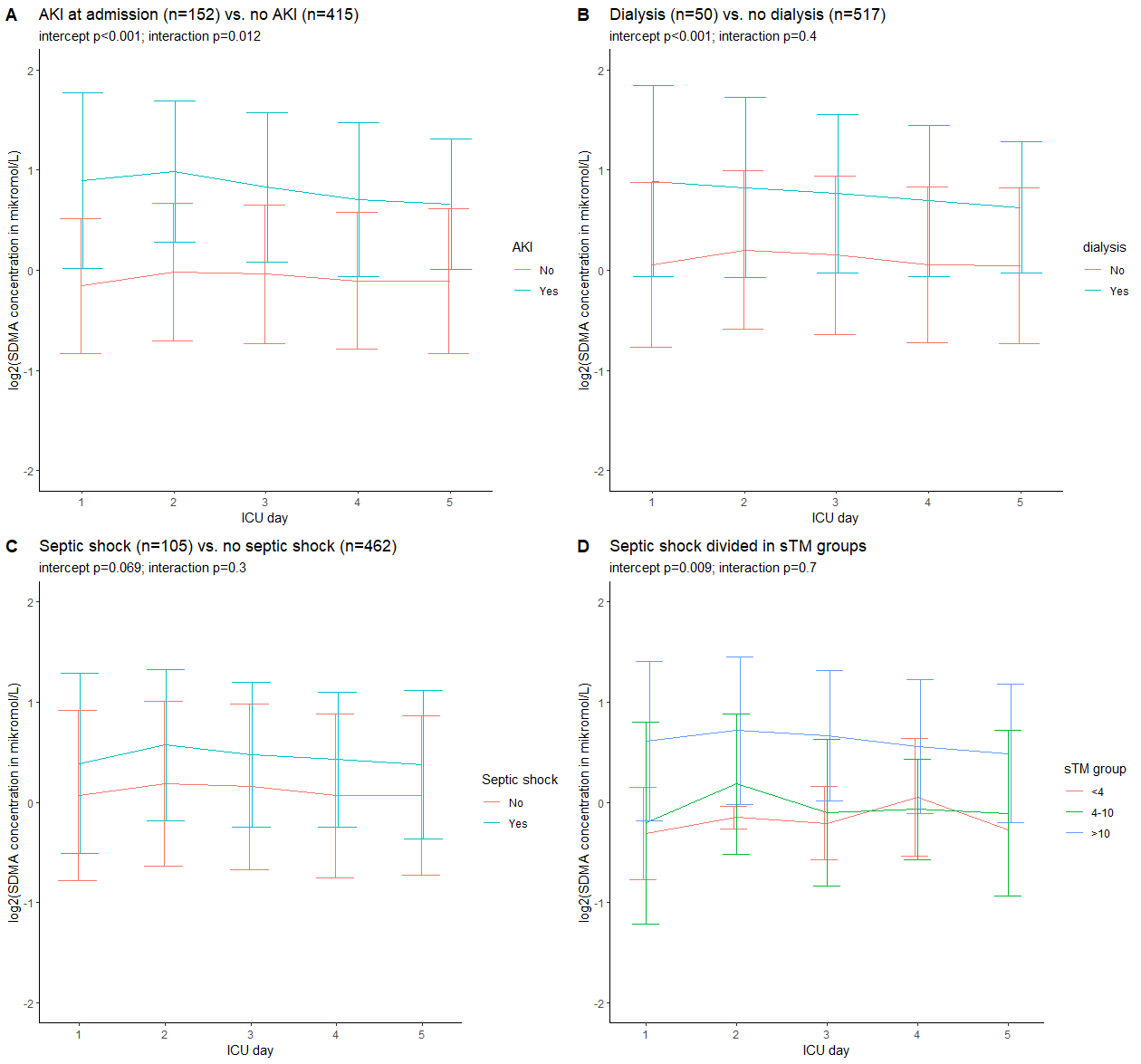
**

Figures show mean +/- sd. AKI is defined as a KDIGO(34) score ≥2 at admission. Dialysis is defined as treatment with continuous renal replacement therapy (CRRT) or hemodialysis (HD) on any day during the 5-day study period. Septic shock is defined as a suspected or proven infection with a need for vasopressor treatment and plasma lactate ≥ 2 mmol/L at admission. sTM groups are defined as sTM <4 ng/mL (n=5) versus 4-10 ng/mL (n=21) versus >10 ng/mL (n=77). SDMA = symmetric dimethylarginine, sTM = soluble thrombomodulin.

**Supplemental Table 5:** SDMA concentrations in patients with AKI at admission (n=152) versus patients without AKI (n=415)

| **Variable** | **estimate** | **95% CI**^1^ | **p-value** |
| --- | --- | --- | --- |
| Sample time |  |  | <0.001 |
| ICU day 1-2 | 0.12 | 0.07, 0.18 |  |
| ICU day 2-3 | -0.04 | -0.10, 0.02 |  |
| ICU day 3-4 | -0.09 | -0.16, -0.03 |  |
| ICU day 4-5 | -0.01 | -0.09, 0.07 |  |
| AKI^2^ | 1.1 | 0.86, 1.3 | <0.001 |
| Sample time * AKI |  |  | 0.012 |
| ICU day 1-2 * AKI | -0.03 | -0.13, 0.08 |  |
| ICU day 2-3 * AKI | -0.09 | -0.20, 0.02 |  |
| ICU day 3-4 * AKI | -0.01 | -0.13, 0.11 |  |
| ICU day 4-5 * AKI | -0.08 | -0.22, 0.06 |  |
| ^1^CI = Confidence Interval | | | |
| ^2^KDIGO score ≥2 at admission | | | |

**Supplemental Table 6:** SDMA concentrations in patients treated with dialysis at any day during the five day study period (n=50) versus patients not treated with dialysis (n=517)

| **Variable** | **estimate** | **95% CI**^1^ | **p-value** |
| --- | --- | --- | --- |
| Sample time |  |  | <0.001 |
| ICU day 1-2 | 0.13 | 0.08, 0.18 |  |
| ICU day 2-3 | -0.07 | -0.12, -0.01 |  |
| ICU day 3-4 | -0.11 | -0.17, -0.05 |  |
| ICU day 4-5 | -0.03 | -0.10, 0.04 |  |
| Dialysis^2^ | 0.93 | 0.60, 1.3 | <0.001 |
| Sample time * Dialysis |  |  | 0.4 |
| ICU day 1-2 * Dialysis | -0.14 | -0.30, 0.02 |  |
| ICU day 2-3 * Dialysis | 0.02 | -0.14, 0.18 |  |
| ICU day 3-4 * Dialysis | 0.07 | -0.09, 0.24 |  |
| ICU day 4-5 * Dialysis | -0.06 | -0.24, 0.13 |  |
| ^1^CI = Confidence Interval | | | |
| ^2^at any day during the first five days of ICU stay | | | |

**Supplemental Table 7:** SDMA concentrations in patients with septic shock (n=105) versus patients without septic shock (n=462)

| **Variable** | **estimate** | **95% CI**^1^ | **p-value** |
| --- | --- | --- | --- |
| Sample time |  |  | <0.001 |
| ICU day 1-2 | 0.10 | 0.05, 0.15 |  |
| ICU day 2-3 | -0.07 | -0.12, -0.01 |  |
| ICU day 3-4 | -0.11 | -0.17, -0.05 |  |
| ICU day 4-5 | -0.03 | -0.10, 0.05 |  |
| Septic Shock^2^ | 0.23 | -0.02, 0.47 | 0.069 |
| Sample time * Septic Shock |  |  | 0.3 |
| ICU day 1-2 * Septic Shock | 0.09 | -0.03, 0.21 |  |
| ICU day 2-3 * Septic Shock | 0.00 | -0.12, 0.13 |  |
| ICU day 3-4 * Septic Shock | 0.05 | -0.08, 0.19 |  |
| ICU day 4-5 * Septic Shock | -0.04 | -0.20, 0.11 |  |
| ^1^CI = Confidence Interval | | | |
| ^2^Defined as suspected or proven infection with need for vasopressor treatment and plasma lactate ≥ 2 mmol/L | | | |

**Supplemental Table 8:** SDMA concentrations in patients with septic shock and admission plasma sTM concentration of <4 (n=5) versus 4-10 (n=21) versus >10 ng/mL (n=77)

| **Variable** | **estimate** | **95% CI**^1^ | **p-value** |
| --- | --- | --- | --- |
| Sample time |  |  | 0.8 |
| ICU day 1-2 | 0.12 | -0.44, 0.68 |  |
| ICU day 2-3 | -0.06 | -0.62, 0.50 |  |
| ICU day 3-4 | 0.26 | -0.31, 0.82 |  |
| ICU day 4-5 | -0.09 | -1.0, 0.86 |  |
| sTM group^2^ |  |  | 0.009 |
| <4 | — | — |  |
| 4-10 | 0.04 | -1.1, 1.2 |  |
| >10 | 0.84 | -0.21, 1.9 |  |
| Sample time * sTM group |  |  | 0.7 |
| ICU day 1-2 * sTM group 4-10 | 0.12 | -0.49, 0.74 |  |
| ICU day 2-3 * sTM group 4-10 | -0.18 | -0.82, 0.45 |  |
| ICU day 3-4 * sTM group 4-10 | -0.36 | -1.0, 0.29 |  |
| ICU day 4-5 * sTM group 4-10 | 0.27 | -0.76, 1.3 |  |
| ICU day 1-2 * sTM group >10 | 0.05 | -0.52, 0.62 |  |
| ICU day 2-3 * sTM group >10 | 0.05 | -0.54, 0.63 |  |
| ICU day 3-4 * sTM group >10 | -0.35 | -0.93, 0.24 |  |
| ICU day 4-5 * sTM group >10 | -0.02 | -0.98, 0.95 |  |
| ^1^CI = Confidence Interval | | | |
| ^2^sTM = soluble thrombomodulin (ng/mL) | | | |

1. **Arginine**

**Supplemental Figure 3:** Changes in arginine concentration days 1-5 in subgroups

**
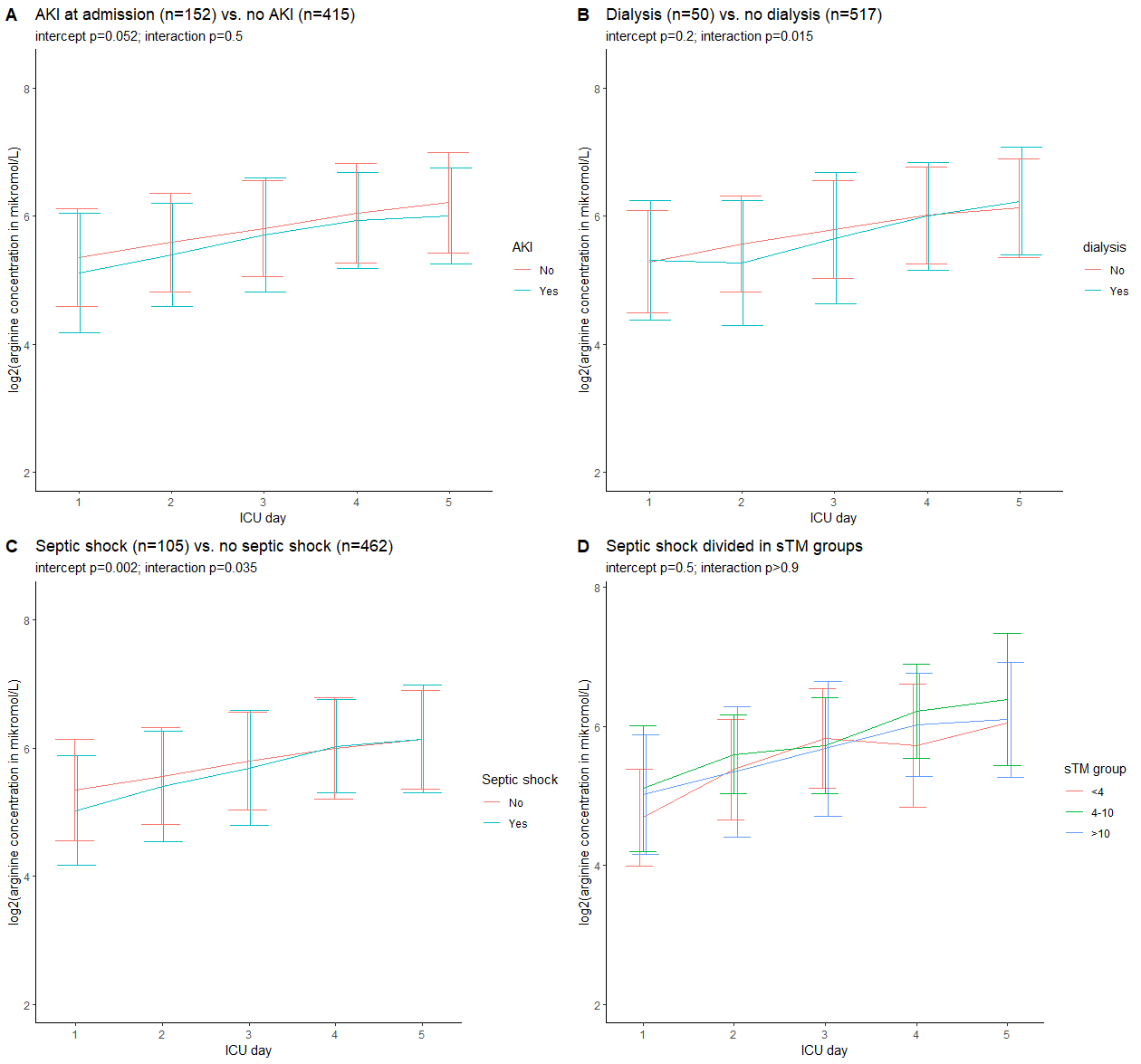
**

Figures show mean +/- sd. AKI is defined as a KDIGO(34) score ≥2 at admission. Dialysis is defined as treatment with continuous renal replacement therapy (CRRT) or hemodialysis (HD) on any day during the 5-day study period. Septic shock is defined as a suspected or proven infection with a need for vasopressor treatment and plasma lactate ≥ 2 mmol/L at admission. sTM groups are defined as sTM <4 ng/mL (n=5) versus 4-10 ng/mL (n=21) versus >10 ng/mL (n=77). sTM = soluble thrombomodulin.

**Supplemental Table 9:** Arginine concentrations in patients with AKI at admission (n=152) versus patients without AKI (n=415)

| **Variable** | **estimate** | **95% CI**^1^ | **p-value** |
| --- | --- | --- | --- |
| Sample time |  |  | <0.001 |
| ICU day 1-2 | 0.24 | 0.15, 0.32 |  |
| ICU day 2-3 | 0.25 | 0.15, 0.34 |  |
| ICU day 3-4 | 0.22 | 0.12, 0.32 |  |
| ICU day 4-5 | 0.18 | 0.07, 0.30 |  |
| AKI^2^ | -0.26 | -0.53, 0.00 | 0.052 |
| Sample time * AKI |  |  | 0.5 |
| ICU day 1-2 * AKI | 0.02 | -0.13, 0.18 |  |
| ICU day 2-3 * AKI | 0.10 | -0.07, 0.26 |  |
| ICU day 3-4 * AKI | 0.00 | -0.18, 0.18 |  |
| ICU day 4-5 * AKI | -0.07 | -0.28, 0.14 |  |
| ^1^CI = Confidence Interval | | | |
| ^2^KDIGO score ≥2 at admission | | | |

**Supplemental Table 10:** Arginine concentrations in patients treated with dialysis at any day during the five day study period (n=50) versus patients not treated with dialysis (n=517)

| **Variable** | **estimate** | **95% CI**^1^ | **p-value** |
| --- | --- | --- | --- |
| Sample time |  |  | <0.001 |
| ICU day 1-2 | 0.27 | 0.20, 0.34 |  |
| ICU day 2-3 | 0.26 | 0.18, 0.34 |  |
| ICU day 3-4 | 0.20 | 0.11, 0.29 |  |
| ICU day 4-5 | 0.14 | 0.03, 0.25 |  |
| Dialysis^2^ | 0.27 | -0.15, 0.69 | 0.2 |
| Sample time * Dialysis |  |  | 0.015 |
| ICU day 1-2 * Dialysis | -0.29 | -0.53, -0.05 |  |
| ICU day 2-3 * Dialysis | 0.14 | -0.10, 0.38 |  |
| ICU day 3-4 * Dialysis | 0.17 | -0.08, 0.41 |  |
| ICU day 4-5 * Dialysis | 0.11 | -0.16, 0.38 |  |
| ^1^CI = Confidence Interval | | | |
| ^2^at any day during the first five days of ICU stay | | | |

**Supplemental Table 11:** Arginine concentrations in patients with septic shock (n=105) versus patients without septic shock (n=462)

| **Variable** | **estimate** | **95% CI**^1^ | **p-value** |
| --- | --- | --- | --- |
| Sample time |  |  | <0.001 |
| ICU day 1-2 | 0.21 | 0.13, 0.29 |  |
| ICU day 2-3 | 0.26 | 0.18, 0.35 |  |
| ICU day 3-4 | 0.20 | 0.11, 0.30 |  |
| ICU day 4-5 | 0.16 | 0.05, 0.28 |  |
| Septic Shock^2^ | -0.49 | -0.79, -0.19 | 0.002 |
| Sample time * Septic Shock |  |  | 0.035 |
| ICU day 1-2 * Septic Shock | 0.16 | -0.02, 0.34 |  |
| ICU day 2-3 * Septic Shock | 0.04 | -0.14, 0.23 |  |
| ICU day 3-4 * Septic Shock | 0.09 | -0.12, 0.29 |  |
| ICU day 4-5 * Septic Shock | -0.02 | -0.26, 0.22 |  |
| ^1^CI = Confidence Interval | | | |
| ^2^Defined as suspected or proven infection with need for vasopressor treatment and plasma lactate ≥ 2 mmol/L | | | |

**Supplemental Table 12:** Arginine concentrations in patients with septic shock and admission plasma sTM concentration of <4 (n=5) versus 4-10 (n=21) versus >10 ng/mL (n=77)

| **Variable** | **estimate** | **95% CI**^1^ | **p-value** |
| --- | --- | --- | --- |
| Sample time |  |  | 0.013 |
| ICU day 1-2 | 0.78 | -0.05, 1.6 |  |
| ICU day 2-3 | 0.45 | -0.40, 1.3 |  |
| ICU day 3-4 | -0.10 | -0.96, 0.75 |  |
| ICU day 4-5 | 0.59 | -0.83, 2.0 |  |
| sTM group^2^ |  |  | 0.5 |
| <4 | — | — |  |
| 4-10 | 0.80 | -0.70, 2.3 |  |
| >10 | 0.77 | -0.63, 2.2 |  |
| Sample time * sTM group |  |  | >0.9 |
| ICU day 1-2 * sTM group 4-10 | -0.37 | -1.3, 0.55 |  |
| ICU day 2-3 * sTM group 4-10 | -0.31 | -1.3, 0.64 |  |
| ICU day 3-4 * sTM group 4-10 | 0.55 | -0.43, 1.5 |  |
| ICU day 4-5 * sTM group 4-10 | -0.45 | -2.0, 1.1 |  |
| ICU day 1-2 * sTM group >10 | -0.45 | -1.3, 0.41 |  |
| ICU day 2-3 * sTM group >10 | -0.09 | -0.97, 0.79 |  |
| ICU day 3-4 * sTM group >10 | 0.39 | -0.49, 1.3 |  |
| ICU day 4-5 * sTM group >10 | -0.48 | -1.9, 0.97 |  |
| ^1^CI = Confidence Interval | | | |
| ^2^sTM = soluble thrombomodulin (ng/mL) | | | |

1. **Homoarginine**

**Supplemental Figure 4:** Changes in homoarginine concentration days 1-5 in subgroups

**
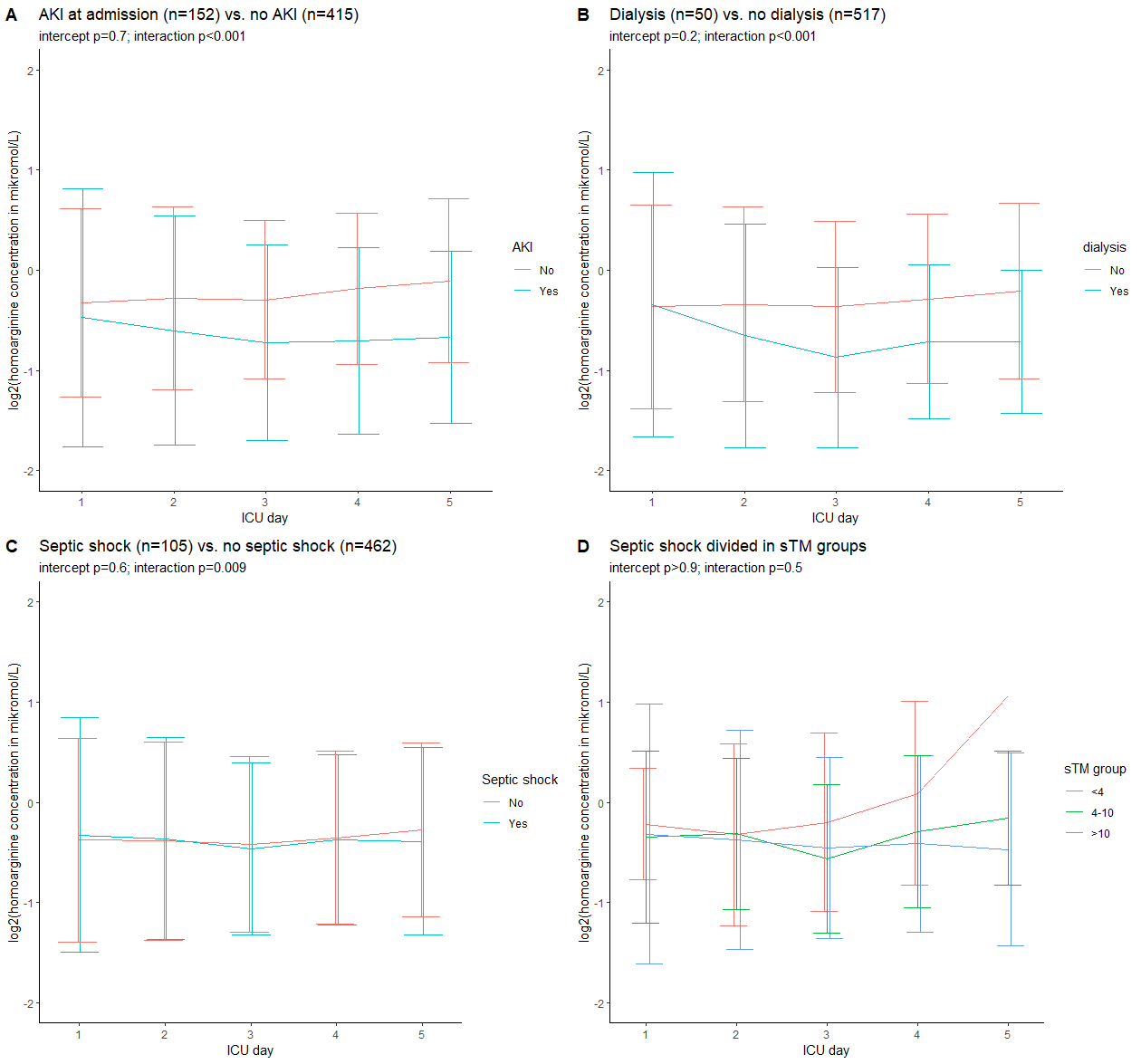
**

Figures show mean +/- sd. AKI is defined as a KDIGO(34) score ≥2 at admission. Dialysis is defined as treatment with continuous renal replacement therapy (CRRT) or hemodialysis (HD) on any day during the 5-day study period. Septic shock is defined as a suspected or proven infection with a need for vasopressor treatment and plasma lactate ≥ 2 mmol/L at admission. sTM groups are defined as sTM <4 ng/mL (n=5) versus 4-10 ng/mL (n=21) versus >10 ng/mL (n=77). sTM = soluble thrombomodulin.

**Supplemental Table 13:** Homoarginine concentrations in patients with AKI at admission (n=152) versus patients without AKI (n=415)

| **Variable** | **estimate** | **95% CI**^1^ | **p-value** |
| --- | --- | --- | --- |
| Sample time |  |  | <0.001 |
| ICU day 1-2 | 0.04 | -0.02, 0.11 |  |
| ICU day 2-3 | 0.04 | -0.03, 0.11 |  |
| ICU day 3-4 | 0.05 | -0.02, 0.13 |  |
| ICU day 4-5 | 0.10 | 0.01, 0.20 |  |
| AKI^2^ | 0.05 | -0.20, 0.29 | 0.7 |
| Sample time * AKI |  |  | <0.001 |
| ICU day 1-2 * AKI | -0.17 | -0.29, -0.04 |  |
| ICU day 2-3 * AKI | -0.11 | -0.24, 0.02 |  |
| ICU day 3-4 * AKI | 0.01 | -0.13, 0.15 |  |
| ICU day 4-5 * AKI | -0.14 | -0.30, 0.03 |  |
| ^1^CI = Confidence Interval | | | |
| ^2^KDIGO score ≥2 at admission | | | |

**Supplemental Table 14:** Homoarginine concentrations in patients treated with dialysis at any day during the five day study period (n=50) versus patients not treated with dialysis (n=517)

| **Variable** | **estimate** | **95% CI**^1^ | **p-value** |
| --- | --- | --- | --- |
| Sample time |  |  | <0.001 |
| ICU day 1-2 | 0.02 | -0.03, 0.08 |  |
| ICU day 2-3 | 0.03 | -0.04, 0.09 |  |
| ICU day 3-4 | 0.06 | -0.01, 0.13 |  |
| ICU day 4-5 | 0.08 | 0.00, 0.16 |  |
| Dialysis^2^ | 0.28 | -0.11, 0.67 | 0.2 |
| Sample time * Dialysis |  |  | <0.001 |
| ICU day 1-2 * Dialysis | -0.29 | -0.48, -0.10 |  |
| ICU day 2-3 * Dialysis | -0.17 | -0.36, 0.02 |  |
| ICU day 3-4 * Dialysis | 0.02 | -0.17, 0.22 |  |
| ICU day 4-5 * Dialysis | -0.11 | -0.33, 0.10 |  |
| ^1^CI = Confidence Interval | | | |
| ^2^at any day during the first five days of ICU stay | | | |

**Supplemental Table 15:** Homoarginine concentrations in patients with septic shock (n=105) versus patients without septic shock (n=462)

| **Variable** | **estimate** | **95% CI**^1^ | **p-value** |
| --- | --- | --- | --- |
| Sample time |  |  | <0.001 |
| ICU day 1-2 | 0.01 | -0.05, 0.07 |  |
| ICU day 2-3 | 0.02 | -0.05, 0.09 |  |
| ICU day 3-4 | 0.06 | -0.01, 0.14 |  |
| ICU day 4-5 | 0.09 | 0.00, 0.18 |  |
| Septic Shock^2^ | 0.09 | -0.20, 0.37 | 0.6 |
| Sample time * Septic Shock |  |  | 0.009 |
| ICU day 1-2 * Septic Shock | -0.05 | -0.20, 0.09 |  |
| ICU day 2-3 * Septic Shock | -0.09 | -0.24, 0.06 |  |
| ICU day 3-4 * Septic Shock | -0.03 | -0.19, 0.13 |  |
| ICU day 4-5 * Septic Shock | -0.14 | -0.33, 0.05 |  |
| ^1^CI = Confidence Interval | | | |
| ^2^Defined as suspected or proven infection with need for vasopressor treatment and plasma lactate ≥ 2 mmol/L | | | |

**Supplemental Table 16:** Homoarginine concentrations in patients with septic shock and admission plasma sTM concentration of <4 (n=5) versus 4-10 (n=21) versus >10 ng/mL (n=77)

| **Variable** | **estimate** | **95% CI**^1^ | **p-value** |
| --- | --- | --- | --- |
| Sample time |  |  | 0.7 |
| ICU day 1-2 | 0.00 | -0.72, 0.73 |  |
| ICU day 2-3 | 0.12 | -0.61, 0.86 |  |
| ICU day 3-4 | 0.29 | -0.45, 1.0 |  |
| ICU day 4-5 | 0.03 | -1.2, 1.3 |  |
| sTM group^2^ |  |  | >0.9 |
| <4 | — | — |  |
| 4-10 | -0.17 | -1.7, 1.3 |  |
| >10 | -0.03 | -1.4, 1.3 |  |
| Sample time * sTM group |  |  | 0.5 |
| ICU day 1-2 * sTM group 4-10 | 0.00 | -0.80, 0.79 |  |
| ICU day 2-3 * sTM group 4-10 | -0.34 | -1.2, 0.49 |  |
| ICU day 3-4 * sTM group 4-10 | -0.18 | -1.0, 0.66 |  |
| ICU day 4-5 * sTM group 4-10 | 0.29 | -1.1, 1.6 |  |
| ICU day 1-2 * sTM group >10 | -0.07 | -0.82, 0.67 |  |
| ICU day 2-3 * sTM group >10 | -0.17 | -0.92, 0.59 |  |
| ICU day 3-4 * sTM group >10 | -0.29 | -1.0, 0.47 |  |
| ICU day 4-5 * sTM group >10 | -0.13 | -1.4, 1.1 |  |
| ^1^CI = Confidence Interval | | | |
| ^2^sTM = soluble thrombomodulin (ng/mL) | | | |
