# Supplementary material for: Changes in nitric oxide inhibitors and mortality in critically ill patients: a cohort study
Source: Ann Intensive Care. 2024 Aug 27;14:133. doi: 10.1186/s13613-024-01362-7 (PMC11349968; doi:10.1186/s13613-024-01362-7)
Supplement: Supplementary file 4 — Supplementary Material 4 [file 13613_2024_1362_MOESM4_ESM.docx]

**Additional File 4:** Cox regression – Additional results

**Supplemental Table 17:** ADMA change day 1-3 and 30-day all-cause mortality, n = 512

|  | HR (95% CI) | P value |
| --- | --- | --- |
| ADMA change day 1-3^*^ | 0.45 (0.21-0.98) | 0.046 |
| Age | 1.03 (1.02-1.05) | <0.001 |
| Sex, Male | 1.46 (0.99-2.13) | 0.053 |
| History of CVD^#^ | 1.06 (0.70-1.59) | 0.78 |
| History of diabetes | 0.88 (0.58-1.36) | 0.57 |
| History of hypertension | 0.82 (0.57-1.19) | 0.31 |
| Kidneyfail group “AKI without CKD” | 0.98 (0.53-1.84) | 0.96 |
| Kidneyfail group “CKD without AKI” | 1.69 (0.89-3.20) | 0.11 |
| Kidneyfail group “AKI and CKD” | 0.67 (0.31-1.47) | 0.32 |
| MELD spline 1^$^ | 0.98 (0.89-1.07) | 0.61 |
| MELD spline 2^$^ | 1.12 (0.97-1.31) | 0.13 |

*The change from days 1-3 was estimated as a slope from a linear model for each patient. ADMA concentration was modelled with log2.

^#^History of cardiovascular disease (CVD) were defined as a history of heart failure, myocardial infarction, or stroke.

^$^The MELD score was modelled as a three-knot cubic spline.

HR = hazard ratio, CI = confidence interval, ADMA = asymmetric dimethylarginine, MELD = model for end-stage liver disease.

**Supplemental Table 18:** SDMA change day 1-3 and 30-day all-cause mortality, n = 512

|  | HR (95% CI) | P value |
| --- | --- | --- |
| SDMA change day 1-3^*^ | 1.07 (0.53-2.17) | 0.84 |
| Age | 1.04 (1.02-1.05) | <0.001 |
| Sex, Male | 1.48 (1.01-2.16) | 0.045 |
| History of CVD^#^ | 1.03 (0.69-1.55) | 0.89 |
| History of diabetes | 0.90 (0.59-1.37) | 0.62 |
| History of hypertension | 0.81 (0.56-1.17) | 0.26 |
| Kidneyfail group “AKI without CKD” | 0.96 (0.52-1.78) | 0.89 |
| Kidneyfail group “CKD without AKI” | 1.70 (0.90-3.21) | 0.12 |
| Kidneyfail group “AKI and CKD” | 0.70 (0.32-1.52) | 0.37 |
| MELD spline 1^$^ | 0.97 (0.89-1.06) | 0.54 |
| MELD spline 2^$^ | 1.14 (0.98-1.32) | 0.10 |

*The change from days 1-3 was estimated as a slope from a linear model for each patient. SDMA concentration was modelled with log2.

^#^History of cardiovascular disease (CVD) were defined as a history of heart failure, myocardial infarction, or stroke.

^$^The MELD score was modelled as a three-knot cubic spline.

HR = hazard ratio, CI = confidence interval, SDMA = symmetric dimethylarginine, MELD = model for end-stage liver disease.

**Supplemental Table 19:** Arginine change day 1-3 and 30-day all-cause mortality, n = 512

|  | HR (95% CI) | P value |
| --- | --- | --- |
| Arginine change day 1-3^*^ | 0.70 (0.44-1.12) | 0.14 |
| Age | 1.04 (1.02-1.05) | <0.001 |
| Sex, Male | 1.48 (1.01-2.16) | 0.046 |
| History of CVD^#^ | 1.03 (0.69-1.55) | 0.87 |
| History of diabetes | 0.91 (0.59-1.39) | 0.65 |
| History of hypertension | 0.83 (0.57-1.20) | 0.31 |
| Kidneyfail group “AKI without CKD” | 1.00 (0.53-1.87) | 1.00 |
| Kidneyfail group “CKD without AKI” | 1.68 (0.88-3.18) | 0.12 |
| Kidneyfail group “AKI and CKD” | 0.69 (0.32-1.51) | 0.36 |
| MELD spline 1^$^ | 0.97 (0.89-1.06) | 0.55 |
| MELD spline 2^$^ | 1.13 (0.98-1.32) | 0.10 |

*The change from days 1-3 was estimated as a slope from a linear model for each patient. Arginine concentration was modelled with log2.

^#^History of cardiovascular disease (CVD) were defined as a history of heart failure, myocardial infarction, or stroke.

^$^The MELD score was modelled as a three-knot cubic spline.

HR = hazard ratio, CI = confidence interval, MELD = model for end-stage liver disease.

**Supplemental Table 20:** Homoarginine change day 1-3 and 30-day all-cause mortality, n = 512

|  | HR (95% CI) | P value |
| --- | --- | --- |
| Homoarginine change day 1-3^*^ | 0.84 (0.47-1.50) | 0.55 |
| Age | 1.04 (1.02-1.05) | <0.001 |
| Sex, Male | 1.46 (1.00-2.14) | 0.052 |
| History of CVD^#^ | 1.04 (0.69-1.56) | 0.86 |
| History of diabetes | 0.90 (0.59-1.38) | 0.63 |
| History of hypertension | 0.81 (0.56-1.17) | 0.26 |
| Kidneyfail group “AKI without CKD” | 0.94 (0.50-1.75) | 0.84 |
| Kidneyfail group “CKD without AKI” | 1.69 (0.89-3.20) | 0.11 |
| Kidneyfail group “AKI and CKD” | 0.69 (0.32-1.50) | 0.35 |
| MELD spline 1^$^ | 0.97 (0.89-1.06) | 0.55 |
| MELD spline 2^$^ | 1.13 (0.98-1.32) | 0.10 |

*The change from days 1-3 was estimated as a slope from a linear model for each patient. Homoarginine concentration was modelled with log2.

^#^History of cardiovascular disease (CVD) were defined as a history of heart failure, myocardial infarction, or stroke.

^$^The MELD score was modelled as a three-knot cubic spline.

HR = hazard ratio, CI = confidence interval, MELD = model for end-stage liver disease.

**Supplemental Table 21:** ADMA admission and 30-day all-cause mortality, n = 512

|  | HR (95% CI) | P value |
| --- | --- | --- |
| ADMA admission^*^ | 1.78 (1.24-2.57) | 0.0025 |
| Age | 1.03 (1.02-1.05) | <0.001 |
| Sex, Male | 1.46 (1.00-2.14) | 0.050 |
| History of CVD^#^ | 1.06 (0.70-1.60) | 0.78 |
| History of diabetes | 0.88 (0.57-1.34) | 0.55 |
| History of hypertension | 0.85 (0.59-1.23) | 0.40 |
| Kidneyfail group “AKI without CKD” | 0.98 (0.53-1.81) | 0.95 |
| Kidneyfail group “CKD without AKI” | 1.65 (0.89-3.10) | 0.12 |
| Kidneyfail group “AKI and CKD” | 0.71 (0.33-1.53) | 0.38 |
| MELD spline 1^$^ | 0.97 (0.89-1.06) | 0.56 |
| MELD spline 2^$^ | 1.11 (0.96-1.29) | 0.18 |

^*^Modelled with log2

^#^History of cardiovascular disease (CVD) were defined as a history of heart failure, myocardial infarction, or stroke.

^$^The MELD score was modelled as a three-knot cubic spline.

HR = hazard ratio, CI = confidence interval, ADMA = asymmetric dimethylarginine, MELD = model for end-stage liver disease.

**Supplemental Table 22:** SDMA admission and 30-day all-cause mortality, n = 512

|  | HR (95% CI) | P value |
| --- | --- | --- |
| SDMA admission^*^ | 1.41 (1.05-1.90) | 0.024 |
| Age | 1.03 (1.02-1.05) | <0.001 |
| Sex, Male | 1.43 (0.98-2.09) | 0.068 |
| History of CVD^#^ | 1.05 (0.70-1.59) | 0.81 |
| History of diabetes | 0.87 (0.57-1.33) | 0.53 |
| History of hypertension | 0.78 (0.54-1.12) | 0.19 |
| Kidneyfail group “AKI without CKD” | 0.79 (0.41-1.52) | 0.49 |
| Kidneyfail group “CKD without AKI” | 1.41 (0.73-2.72) | 0.31 |
| Kidneyfail group “AKI and CKD” | 0.53 (0.23-1.20) | 0.13 |
| MELD spline 1^$^ | 0.95 (0.87-1.04) | 0.28 |
| MELD spline 2^$^ | 1.16 (1.00-1.35) | 0.060 |

^*^Modelled with log2

^#^History of cardiovascular disease (CVD) were defined as a history of heart failure, myocardial infarction, or stroke.

^$^The MELD score was modelled as a three-knot cubic spline.

HR = hazard ratio, CI = confidence interval, SDMA = symmetric dimethylarginine, MELD = model for end-stage liver disease.

**Supplemental Table 23:** Arginine admission and 30-day all-cause mortality, n = 512

|  | HR (95% CI) | P value |
| --- | --- | --- |
| Arginine admission^*^ | 1.19 (0.94-1.51) | 0.16 |
| Age | 1.04 (1.02-1.05) | <0.001 |
| Sex, Male | 1.45 (0.99-2.11) | 0.059 |
| History of CVD^#^ | 1.05 (0.70-1.58) | 0.82 |
| History of diabetes | 0.89 (0.58-1.36) | 0.60 |
| History of hypertension | 0.82 (0.70-1.19) | 0.30 |
| Kidneyfail group “AKI without CKD” | 1.02 (0.55-1.89) | 0.96 |
| Kidneyfail group “CKD without AKI” | 1.70 (0.90-3.21) | 0.11 |
| Kidneyfail group “AKI and CKD” | 0.73 (0.34-1.59) | 0.43 |
| MELD spline 1^$^ | 0.97 (0.89-1.06) | 0.55 |
| MELD spline 2^$^ | 1.13 (0.97-1.32) | 0.12 |

^*^Modelled with log2

^#^History of cardiovascular disease (CVD) were defined as a history of heart failure, myocardial infarction, or stroke.

^$^The MELD score was modelled as a three-knot cubic spline.

HR = hazard ratio, CI = confidence interval, MELD = model for end-stage liver disease.

**Supplemental Table 24:** Homoarginine admission and 30-day all-cause mortality, n = 512

|  | HR (95% CI) | P value |
| --- | --- | --- |
| Homoarginine admission^*^ | 1.15 (0.97-1.38) | 0.12 |
| Age | 1.04 (1.02-1.06) | <0.001 |
| Sex, Male | 1.40 (0.96-2.06) | 0.086 |
| History of CVD^#^ | 1.06 (0.71-1.59) | 0.78 |
| History of diabetes | 0.90 (0.59-1.39) | 0.63 |
| History of hypertension | 0.81 (0.56-1.17) | 0.27 |
| Kidneyfail group “AKI without CKD” | 0.97 (0.52-1.79) | 0.92 |
| Kidneyfail group “CKD without AKI” | 1.72 (0.91-3.24) | 0.096 |
| Kidneyfail group “AKI and CKD” | 0.75 (0.34-1.62) | 0.46 |
| MELD spline 1^$^ | 0.98 (0.89-1.07) | 0.58 |
| MELD spline 2^$^ | 1.13 (0.97-1.31) | 0.12 |

^*^Modelled with log2

^#^History of cardiovascular disease (CVD) were defined as a history of heart failure, myocardial infarction, or stroke.

^$^The MELD score was modelled as a three-knot cubic spline.

HR = hazard ratio, CI = confidence interval, MELD = model for end-stage liver disease.

**Supplemental Table 25:** NO biomarkers and 30-day mortality – Univariate Cox regressions

All n = 512

|  | Unadjusted HR (95% CI) | P value |
| --- | --- | --- |
| ADMA change day 1-3*^$^ | 0.45 (0.21-0.95) | 0.039 |
| SDMA change day 1-3*^$^ | 1.11 (0.55-2.24) | 0.76 |
| Arginine change day 1-3*^$^ | 0.70 (0.44-1.10) | 0.13 |
| Homoarginine change day 1-3*^$^ | 0.76 (0.43-1.34) | 0.35 |
| ADMA admission^$^ | 1.92 (1.38-2.69) | <0.001 |
| SDMA admission^$^ | 1.50 (1.22-1.83) | <0.001 |
| Arginine admission^$^ | 1.22 (0.97-1.54) | 0.098 |
| Homoarginine admission^$^ | 1.10 (0.93-1.31) | 0.26 |

*The change from days 1-3 was estimated as a slope from a linear model for each patient.

^$^All NO markers (change day 1-3 and admission) were transformed with log2 and analyzed separately in univariate Cox regressions.

NO = nitric oxide, HR = hazard ratio, CI = confidence interval, ADMA = asymmetric dimethylarginine, SDMA = symmetric dimethylarginine.
