# Supplementary material for: Changes in nitric oxide inhibitors and mortality in critically ill patients: a cohort study
Source: Ann Intensive Care. 2024 Aug 27;14:133. doi: 10.1186/s13613-024-01362-7 (PMC11349968; doi:10.1186/s13613-024-01362-7)
Supplement: Supplementary file 5 — Supplementary Material 5 [file 13613_2024_1362_MOESM5_ESM.docx]

**Additional File 5:** Univariate Cox regression in subgroups

**Supplemental Figure 5:** Univariate Cox regression of the association between ADMA change day 1-3 and 30-day mortality in subgroups.


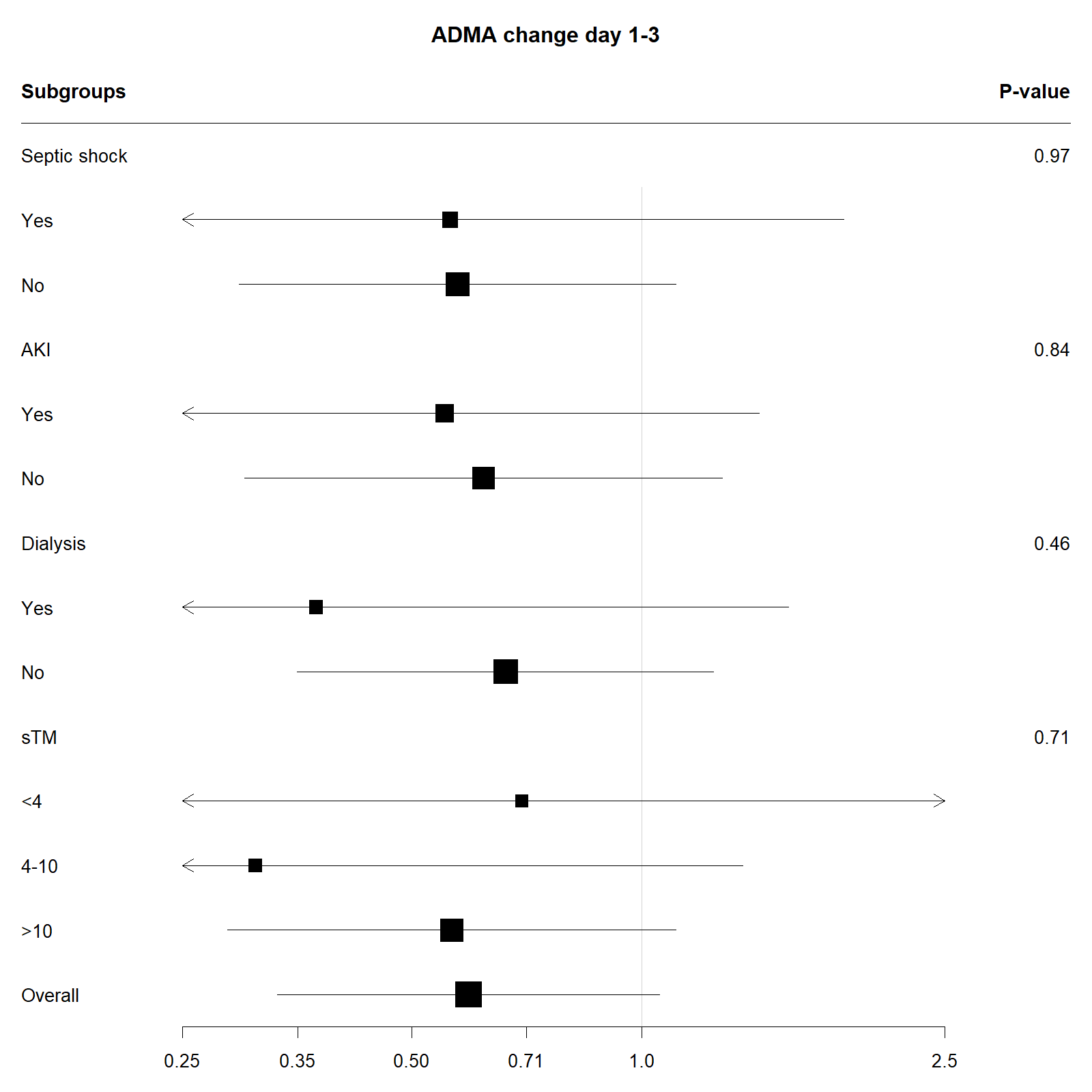


All subgroups are defined by baseline variables. ADMA = asymmetric dimethylarginine, sTM = soluble thrombomodulin.

**Supplemental Figure 6:** Univariate Cox regression of the association between SDMA change day 1-3 and 30-day mortality in subgroups.


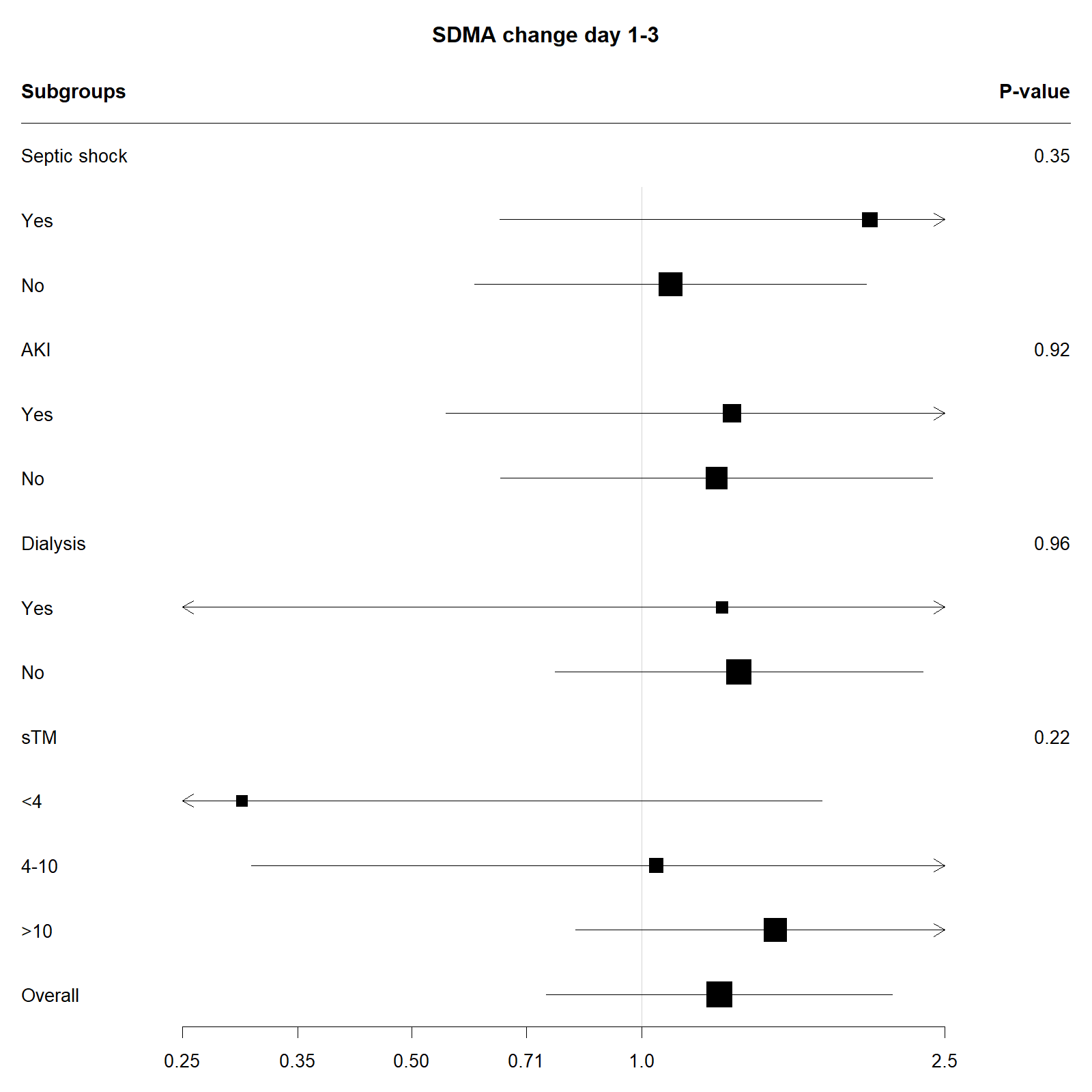


All subgroups are defined by baseline variables. SDMA = symmetric dimethylarginine, sTM = soluble thrombomodulin.

**Supplemental Figure 7:** Univariate Cox regression of the association between arginine change day 1-3 and 30-day mortality in subgroups.


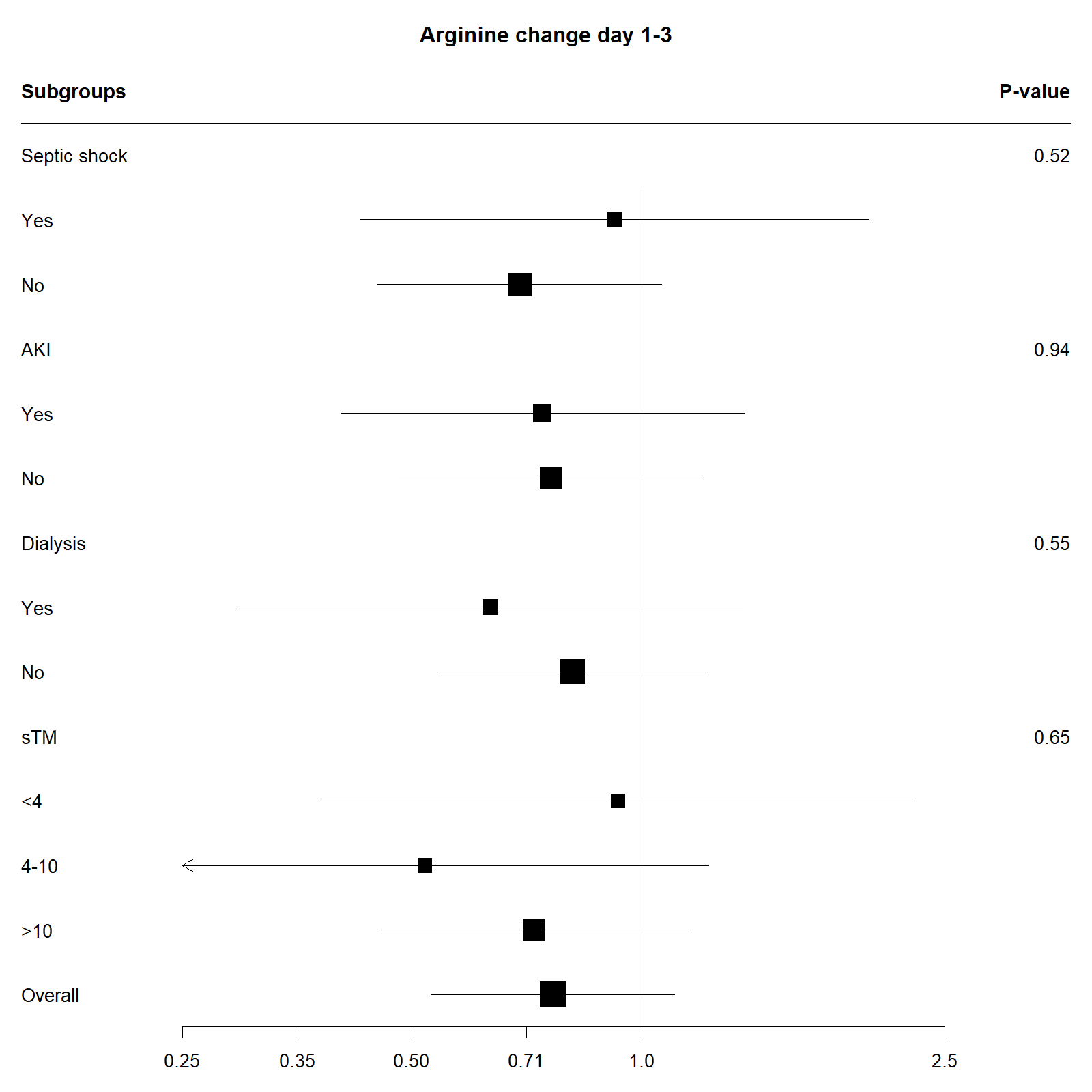


All subgroups are defined by baseline variables. sTM = soluble thrombomodulin.

**Supplemental Figure 8:** Univariate Cox regression of the association between homoarginine change day 1-3 and 30-day mortality in subgroups.


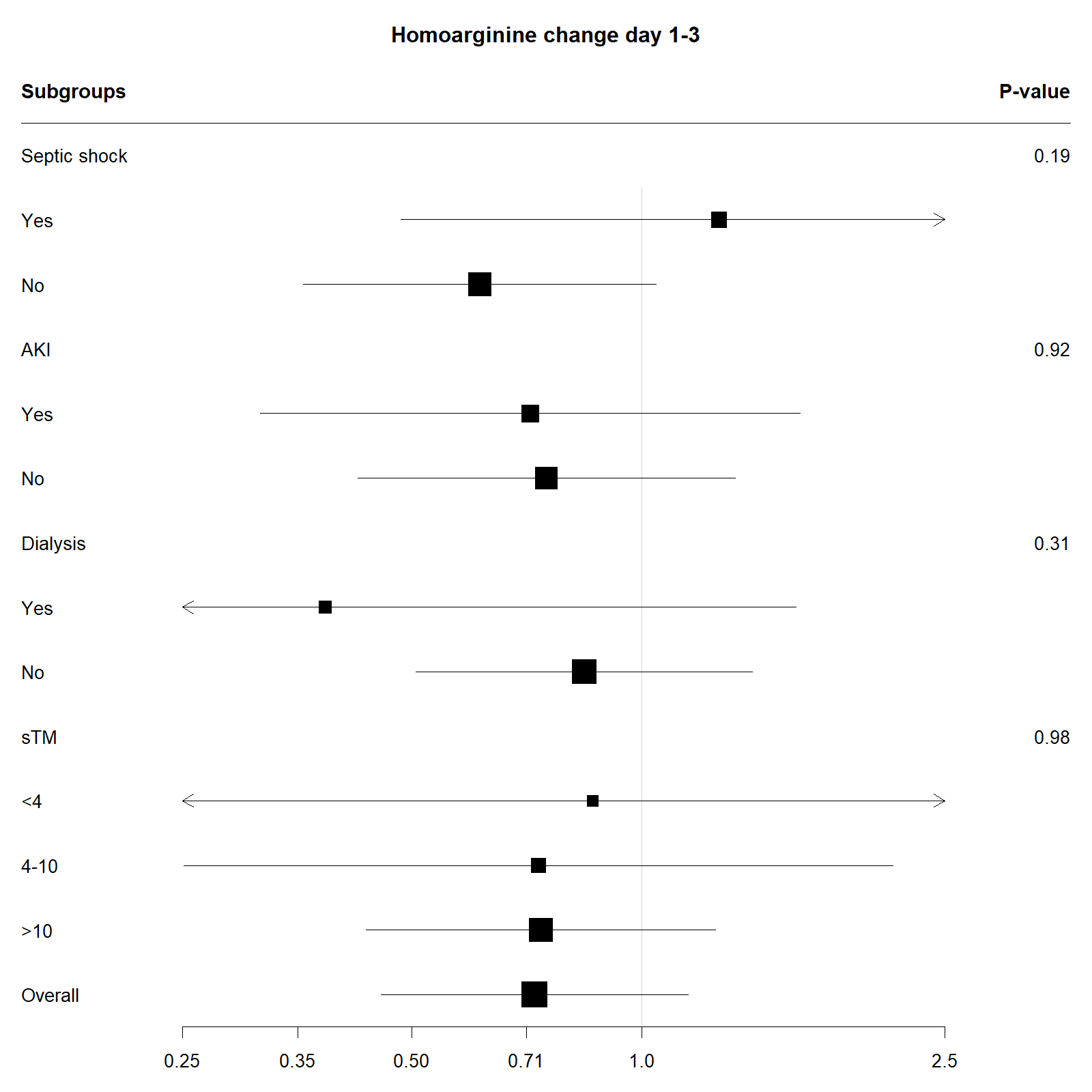


All subgroups are defined by baseline variables. sTM = soluble thrombomodulin.

**Supplemental Figure 9:** Univariate Cox regression of the association between ADMA concentration at admission and 30-day mortality in subgroups.


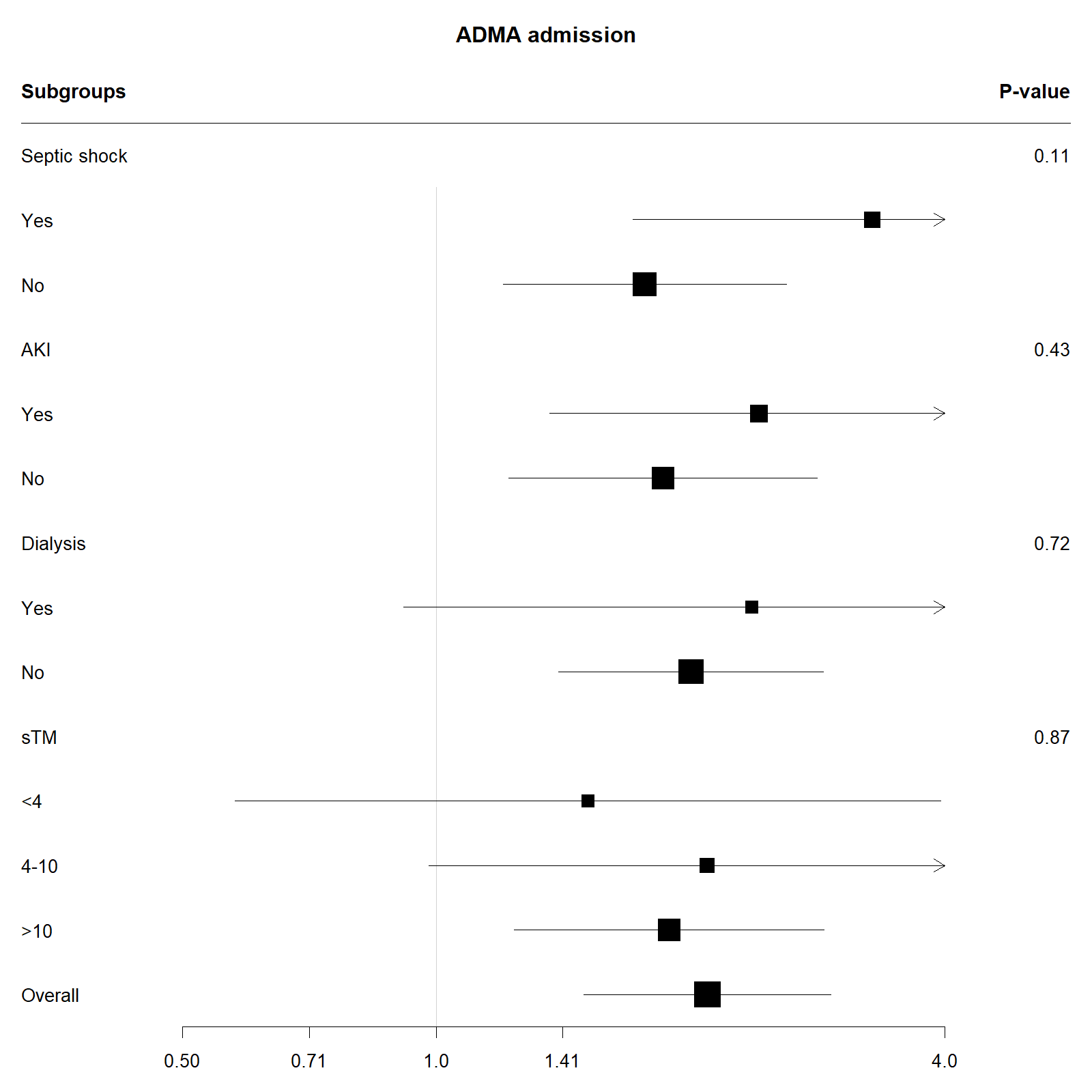


All subgroups are defined by baseline variables. ADMA = asymmetric dimethylarginine, sTM = soluble thrombomodulin.

**Supplemental Figure 10:** Univariate Cox regression of the association between SDMA concentration at admission and 30-day mortality in subgroups.


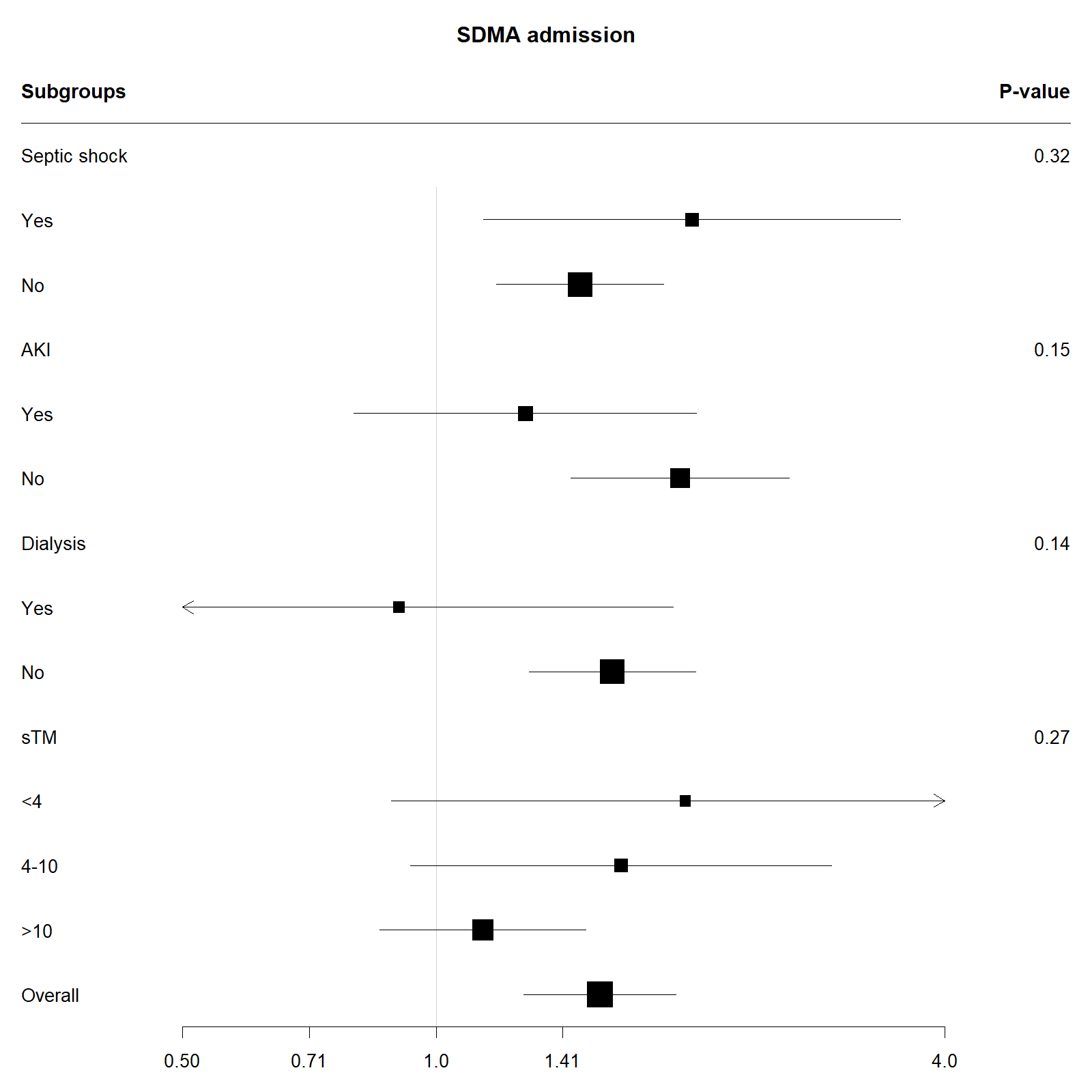


All subgroups are defined by baseline variables. SDMA = symmetric dimethylarginine, sTM = soluble thrombomodulin.

**Supplemental Figure 11:** Univariate Cox regression of the association between arginine concentration at admission and 30-day mortality in subgroups.


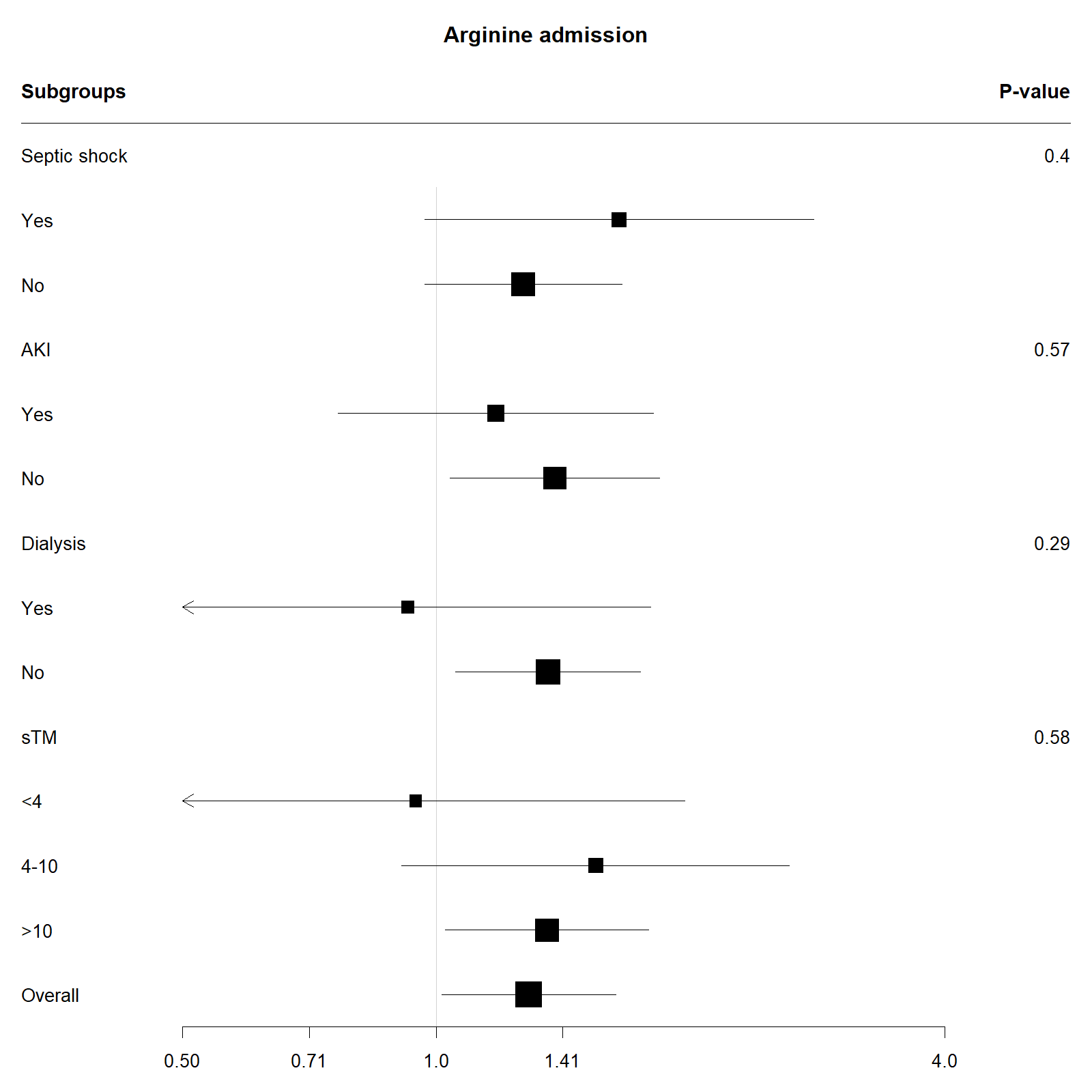


All subgroups are defined by baseline variables. sTM = soluble thrombomodulin.

**Supplemental Figure 12:** Univariate Cox regression of the association between homoarginine concentration at admission and 30-day mortality in subgroups.


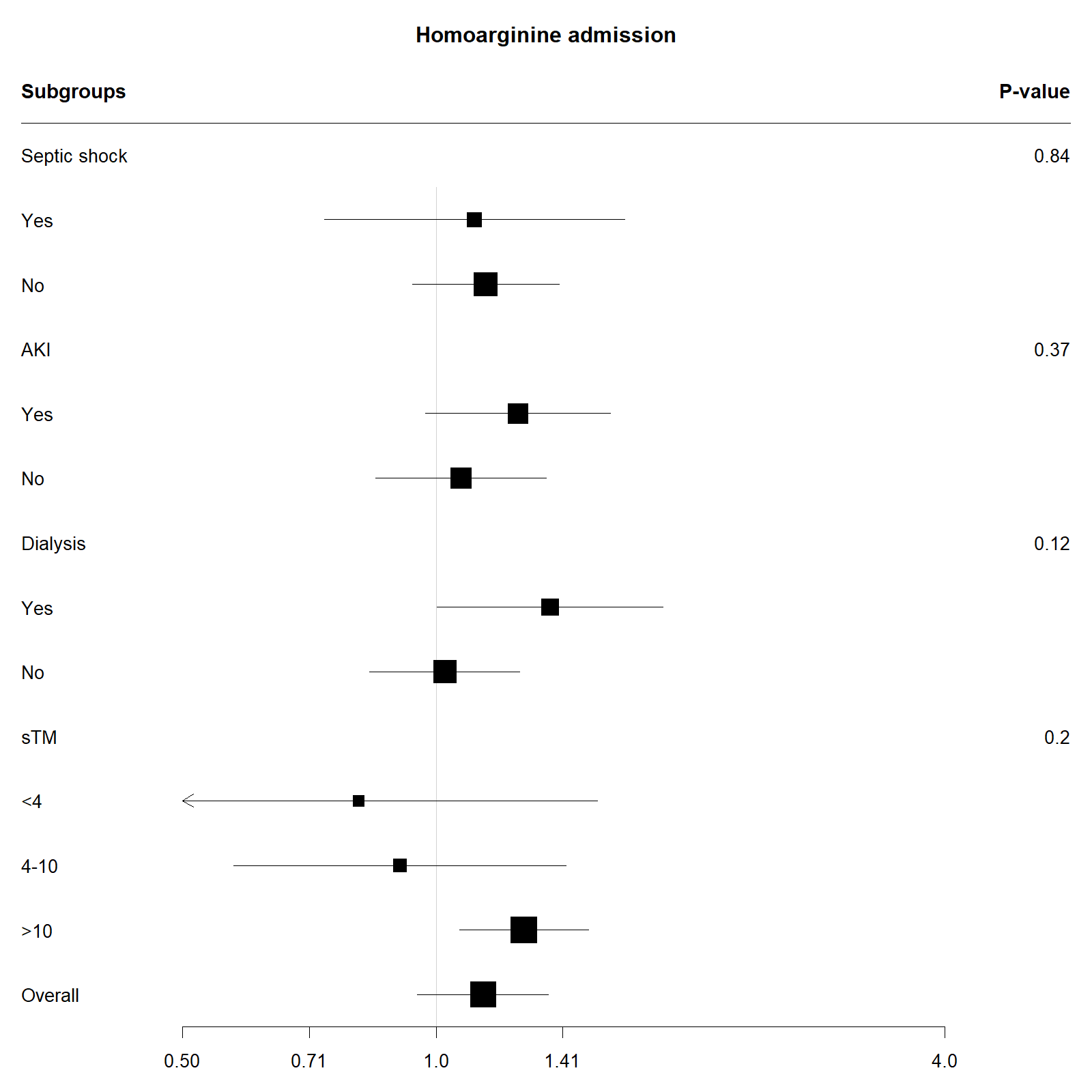


All subgroups are defined by baseline variables. sTM = soluble thrombomodulin.
