# Supplementary material for: Changes in nitric oxide inhibitors and mortality in critically ill patients: a cohort study
Source: Ann Intensive Care. 2024 Aug 27;14:133. doi: 10.1186/s13613-024-01362-7 (PMC11349968; doi:10.1186/s13613-024-01362-7)
Supplement: Supplementary file 6 — Supplementary Material 6 [file 13613_2024_1362_MOESM6_ESM.docx]

**Additional File 6:** Sensitivity analyses

**Supplemental Figure 13:** Overview of missing samples with reasons


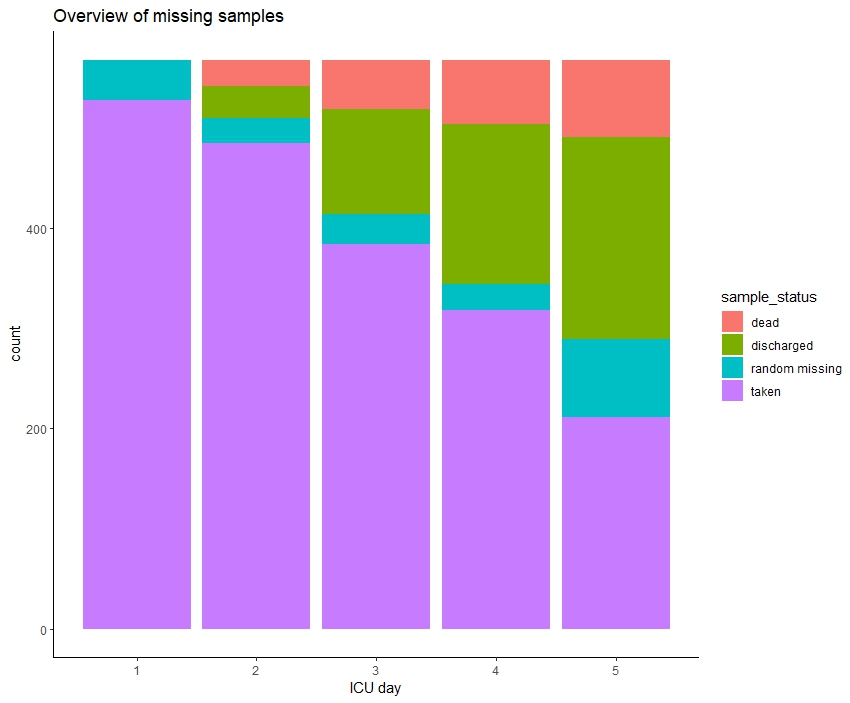


Taken = included in analysis, random missing = patient admitted to ICU but no sample available, discharged = patient discharged from ICU on a previous ICU day, dead = patient died on a previous ICU day

**Supplemental Figure 14:** Sensitivity analyses of changes in NO-biomarkers days 1-5


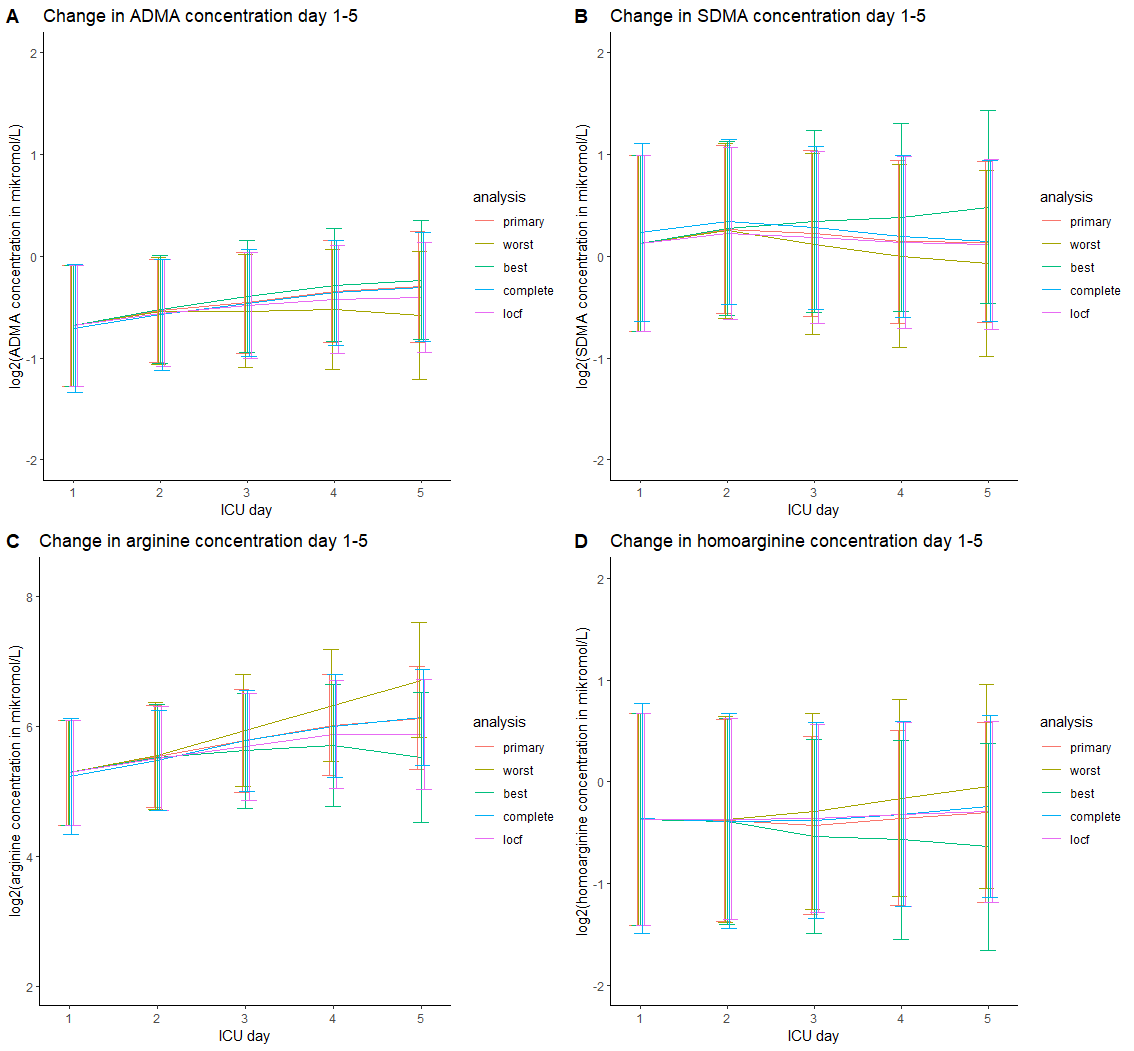


The linear mixed model accounted for missing samples with maximum likelihood inference in the primary analysis (annotated “primary” in the figure). In the worst-case scenario of the worst- and best-case assessment (annotated “worst” in the figure) missing values of patients who died within the five-day study period were assigned the 90^th^ percentile of ADMA and SDMA and the 10^th^ percentile of arginine and homoarginine. Meanwhile missing values of patients discharged from ICU within the study period were assigned the 10^th^ percentile for ADMA and SDMA and the 90^th^ percentile for arginine and homoarginine. In the best-case scenario of the worst- and best-case assessment (annotated “best” in the figure), values were assigned opposite of the worst-case scenario. In complete case analysis (annotated “complete” in the figure) only patients with a complete series of five blood samples were included. In the last observation carried forward analysis (annotated “locf” in figure) the last known value replaced a missing value. ADMA = asymmetric dimethylarginine, SDMA = symmetric dimethylarginine, locf = last observation carried forward.

Sensitivity analyses of multivariate Cox regressions

**Supplemental Table 26:** ADMA change day 1-3 and 30-day mortality in worst-case sensitivity analysis, n = 498

|  | HR (95% CI) | P value |
| --- | --- | --- |
| ADMA change day 1-3* | 3.97 (1.81-8.69) | p=0.001 |
| Age | 1.04 (1.02-1.05) | p<0.001 |
| Sex, Male | 1.53 (1.05-2.25) | p=0.029 |
| History of CVD^#^ | 1.06 (0.70-1.62) | p=0.77 |
| History of diabetes | 0.88 (0.57-1.36) | p=0.56 |
| History of hypertension | 0.75 (0.52-1.08) | p=0.13 |
| Kidneyfail group “AKI without CKD” | 0.96 (0.52-1.78) | p=0.89 |
| Kidneyfail group “CKD without AKI” | 1.71 (0.89-3.28) | p=0.11 |
| Kidneyfail group “AKI and CKD” | 0.77 (0.35-1.72) | p=0.53 |
| MELD spline 1^$^ | 0.94 (0.86-1.04) | p=0.22 |
| MELD spline 2^$^ | 1.21 (1.03-1.44) | p=0.024 |

*The change from day 1-3 was estimated as a slope from a linear model for each patient. ADMA concentration was modelled with log2.

^#^History of cardiovascular disease (CVD) were defined as history of heart failure, myocardial infarction, or stroke.

^$^The MELD score was modelled as a three-knot cubic spline.

HR = hazard ratio, CI = confidence interval, ADMA = asymmetric dimethylarginine, MELD = model for end-stage liver disease.

**Supplemental Table 27:** ADMA change day 1-3 and 30-day mortality in best-case sensitivity analysis, n = 498

|  | HR (95% CI) | P value |
| --- | --- | --- |
| ADMA change day 1-3* | 0.06 (0.02-0.16) | p<0.001 |
| Age | 1.03 (1.02-1.05) | p<0.001 |
| Sex, Male | 1.44 (0.98-2.10) | p=0.061 |
| History of CVD^#^ | 1.05 (0.69-1.58) | p=0.83 |
| History of diabetes | 0.84 (0.54-1.30) | p=0.44 |
| History of hypertension | 0.84 (0.58-1.21) | p=0.35 |
| Kidneyfail group “AKI without CKD” | 0.91 (0.49-1.70) | p=0.77 |
| Kidneyfail group “CKD without AKI” | 1.82 (0.95-3.48) | p=0.072 |
| Kidneyfail group “AKI and CKD” | 0.77 (0.35-1.70) | p=0.52 |
| MELD spline 1^$^ | 0.95 (0.87-1.04) | p=0.26 |
| MELD spline 2^$^ | 1.19 (1.01-1.40) | p=0.035 |

*The change from day 1-3 was estimated as a slope from a linear model for each patient. ADMA concentration was modelled with log2.

^#^History of cardiovascular disease (CVD) were defined as history of heart failure, myocardial infarction, or stroke.

^$^The MELD score was modelled as a three-knot cubic spline.

HR = hazard ratio, CI = confidence interval, ADMA = asymmetric dimethylarginine, MELD = model for end-stage liver disease.

**Supplemental Table 28:** ADMA change day 1-3 and 30-day mortality in complete case sensitivity analysis, n = 340

|  | HR (95% CI) | P value |
| --- | --- | --- |
| ADMA change day 1-3* | 0.32 (0.08-1.24) | p=0.10 |
| Age | 1.03 (1.01-1.06) | p=0.002 |
| Sex, Male | 1.84 (1.17-2.90) | p=0.008 |
| History of CVD^#^ | 0.86 (0.51-1.44) | p=0.56 |
| History of diabetes | 0.71 (0.41-1.25) | p=0.24 |
| History of hypertension | 1.38 (0.86-2.20) | p=0.18 |
| Kidneyfail group “AKI without CKD” | 1.33 (0.55-3.21) | p=0.52 |
| Kidneyfail group “CKD without AKI” | 3.88 (1.90-7.93) | p<0.001 |
| Kidneyfail group “AKI and CKD” | 1.25 (0.42-3.71) | p=0.69 |
| MELD spline 1^$^ | 0.78 (0.70-0.87) | p<0.001 |
| MELD spline 2^$^ | 1.63 (1.36-1.96) | p<0.001 |

*The change from day 1-3 was estimated as a slope from a linear model for each patient. ADMA concentration was modelled with log2.

^#^History of cardiovascular disease (CVD) were defined as history of heart failure, myocardial infarction, or stroke.

^$^The MELD score was modelled as a three-knot cubic spline.

HR = hazard ratio, CI = confidence interval, ADMA = asymmetric dimethylarginine, MELD = model for end-stage liver disease.

**Supplemental Table 29:** ADMA change day 1-3 and 30-day mortality in last observation carried forward sensitivity analysis, n = 503

|  | HR (95% CI) | P value |
| --- | --- | --- |
| ADMA change day 1-3* | 0.47 (0.13-1.69) | p=0.25 |
| Age | 1.03 (1.02-1.05) | p<0.001 |
| Sex, Male | 1.46 (1.00-2.13) | p=0.052 |
| History of CVD^#^ | 1.06 (0.70-1.59) | p=0.80 |
| History of diabetes | 0.90 (0.59-1.39) | p=0.63 |
| History of hypertension | 0.80 (0.55-1.15) | p=0.23 |
| Kidneyfail group “AKI without CKD” | 0.97 (0.52-1.81) | p=0.93 |
| Kidneyfail group “CKD without AKI” | 1.64 (0.86-3.15) | p=0.14 |
| Kidneyfail group “AKI and CKD” | 0.65 (0.29-1.45) | p=0.29 |
| MELD spline 1^$^ | 0.97 (0.89-1.07) | p=0.58 |
| MELD spline 2^$^ | 1.14 (0.97-1.34) | p=0.11 |

*The change from day 1-3 was estimated as a slope from a linear model for each patient. ADMA concentration was modelled with log2.

^#^History of cardiovascular disease (CVD) were defined as history of heart failure, myocardial infarction, or stroke.

^$^The MELD score was modelled as a three-knot cubic spline.

HR = hazard ratio, CI = confidence interval, ADMA = asymmetric dimethylarginine, MELD = model for end-stage liver disease.

**Supplemental Table 30:** SDMA change day 1-3 and 30-day mortality in worst-case sensitivity analysis, n = 498

|  | HR (95% CI) | P value |
| --- | --- | --- |
| SDMA change day 1-3* | 4.56 (2.52-8.25) | p<0.001 |
| Age | 1.04 (1.02-1.05) | p<0.001 |
| Sex, Male | 1.50 (1.02-2.19) | p=0.039 |
| History of CVD^#^ | 1.08 (0.71-1.64) | p=0.73 |
| History of diabetes | 0.79 (0.51-1.23) | p=0.29 |
| History of hypertension | 0.83 (0.57-1.21) | p=0.34 |
| Kidneyfail group “AKI without CKD” | 1.02 (0.55-1.88) | p=0.95 |
| Kidneyfail group “CKD without AKI” | 1.75 (0.91-3.38) | p=0.095 |
| Kidneyfail group “AKI and CKD” | 0.94 (0.42-2.10) | p=0.88 |
| MELD spline 1^$^ | 0.96 (0.88-1.06) | p=0.43 |
| MELD spline 2^$^ | 1.18 (1.00-1.39) | p=0.057 |

*The change from day 1-3 was estimated as a slope from a linear model for each patient. SDMA concentration was modelled with log2.

^#^History of cardiovascular disease (CVD) were defined as history of heart failure, myocardial infarction, or stroke.

^$^The MELD score was modelled as a three-knot cubic spline.

HR = hazard ratio, CI = confidence interval, SDMA = symmetric dimethylarginine, MELD = model for end-stage liver disease.

**Supplemental Table 31:** SDMA change day 1-3 and 30-day mortality in best-case sensitivity analysis, n = 498

|  | HR (95% CI) | P value |
| --- | --- | --- |
| SDMA change day 1-3* | 0.16 (0.08-0.32) | p<0.001 |
| Age | 1.03 (1.02-1.05) | p<0.001 |
| Sex, Male | 1.42 (0.97-2.08) | p=0.072 |
| History of CVD^#^ | 1.05 (0.69-1.59) | p=0.83 |
| History of diabetes | 0.88 (0.57-1.36) | p=0.57 |
| History of hypertension | 0.77 (0.53-1.11) | p=0.16 |
| Kidneyfail group “AKI without CKD” | 0.81 (0.43-1.52) | p=0.51 |
| Kidneyfail group “CKD without AKI” | 1.63 (0.85-3.10) | p=0.14 |
| Kidneyfail group “AKI and CKD” | 0.64 (0.29-1.42) | p=0.28 |
| MELD spline 1^$^ | 0.92 (0.84-1.01) | p=0.080 |
| MELD spline 2^$^ | 1.25 (1.06-1.48) | p=0.008 |

*The change from day 1-3 was estimated as a slope from a linear model for each patient. SDMA concentration was modelled with log2.

^#^History of cardiovascular disease (CVD) were defined as history of heart failure, myocardial infarction, or stroke.

^$^The MELD score was modelled as a three-knot cubic spline.

HR = hazard ratio, CI = confidence interval, SDMA = symmetric dimethylarginine, MELD = model for end-stage liver disease.

**Supplemental Table 32:** SDMA change day 1-3 and 30-day mortality in complete case sensitivity analysis, n = 340

|  | HR (95% CI) | P value |
| --- | --- | --- |
| SDMA change day 1-3* | 0.39 (0.10-1.50) | p=0.17 |
| Age | 1.03 (1.01-1.06) | p=0.002 |
| Sex, Male | 1.91 (1.21-3.02) | p=0.006 |
| History of CVD^#^ | 0.86 (0.51-1.45) | p=0.58 |
| History of diabetes | 0.73 (0.42-1.28) | p=0.28 |
| History of hypertension | 1.31 (0.82-2.10) | p=0.26 |
| Kidneyfail group “AKI without CKD” | 1.25 (0.51-3.02) | p=0.63 |
| Kidneyfail group “CKD without AKI” | 3.60 (1.79-7.24) | p<0.001 |
| Kidneyfail group “AKI and CKD” | 1.06 (0.35-3.16) | p=0.92 |
| MELD spline 1^$^ | 0.77 (0.69-0.86) | p<0.001 |
| MELD spline 2^$^ | 1.66 (1.38-2.00) | p<0.001 |

*The change from day 1-3 was estimated as a slope from a linear model for each patient. SDMA concentration was modelled with log2.

^#^History of cardiovascular disease (CVD) were defined as history of heart failure, myocardial infarction, or stroke.

^$^The MELD score was modelled as a three-knot cubic spline.

HR = hazard ratio, CI = confidence interval, SDMA = symmetric dimethylarginine, MELD = model for end-stage liver disease.

**Supplemental Table 33:** SDMA change day 1-3 and 30-day mortality in last observation carried forward sensitivity analysis, n = 503

|  | HR (95% CI) | P value |
| --- | --- | --- |
| SDMA change day 1-3* | 0.89 (0.28-2.82) | p=0.84 |
| Age | 1.04 (1.02-1.05) | p<0.001 |
| Sex, Male | 1.48 (1.01-2.16) | p=0.043 |
| History of CVD^#^ | 1.05 (0.70-1.59) | p=0.80 |
| History of diabetes | 0.91 (0.59-1.40) | p=0.66 |
| History of hypertension | 0.78 (0.54-1.13) | p=0.20 |
| Kidneyfail group “AKI without CKD” | 0.96 (0.51-1.78) | p=0.89 |
| Kidneyfail group “CKD without AKI” | 1.61 (0.84-3.08) | p=0.15 |
| Kidneyfail group “AKI and CKD” | 0.65 (0.29-1.44) | p=0.29 |
| MELD spline 1^$^ | 0.97 (0.89-1.06) | p=0.52 |
| MELD spline 2^$^ | 1.15 (0.98-1.35) | p=0.094 |

*The change from day 1-3 was estimated as a slope from a linear model for each patient. SDMA concentration was modelled with log2.

^#^History of cardiovascular disease (CVD) were defined as history of heart failure, myocardial infarction, or stroke.

^$^The MELD score was modelled as a three-knot cubic spline.

HR = hazard ratio, CI = confidence interval, SDMA = symmetric dimethylarginine, MELD = model for end-stage liver disease.

**Supplemental Table 34:** Arginine change day 1-3 and 30-day mortality in worst-case sensitivity analysis, n = 498

|  | HR (95% CI) | P value |
| --- | --- | --- |
| Arginine change day 1-3* | 0.25 (0.12-0.52) | p<0.001 |
| Age | 1.03 (1.02-1.05) | p<0.001 |
| Sex, Male | 1.49 (1.02-2.18) | p=0.039 |
| History of CVD^#^ | 1.03 (0.68-1.56) | p=0.88 |
| History of diabetes | 0.92 (0.59-1.43) | p=0.71 |
| History of hypertension | 0.81 (0.56-1.18) | p=0.28 |
| Kidneyfail group “AKI without CKD” | 1.03 (0.55-1.92) | p=0.93 |
| Kidneyfail group “CKD without AKI” | 1.74 (0.91-3.33) | p=0.095 |
| Kidneyfail group “AKI and CKD” | 0.73 (0.33-1.61) | p=0.44 |
| MELD spline 1^$^ | 0.96 (0.87-1.05) | p=0.35 |
| MELD spline 2^$^ | 1.17 (1.00-1.38) | p=0.052 |

*The change from day 1-3 was estimated as a slope from a linear model for each patient. Arginine concentration was modelled with log2.

^#^History of cardiovascular disease (CVD) were defined as history of heart failure, myocardial infarction, or stroke.

^$^The MELD score was modelled as a three-knot cubic spline.

HR = hazard ratio, CI = confidence interval, MELD = model for end-stage liver disease.

**Supplemental Table 35:** Arginine change day 1-3 and 30-day mortality in best-case sensitivity analysis, n = 498

|  | HR (95% CI) | P value |
| --- | --- | --- |
| Arginine change day 1-3* | 2.77 (1.74-4.41) | p<0.001 |
| Age | 1.04 (1.02-1.05) | p<0.001 |
| Sex, Male | 1.50 (1.02-2.19) | p=0.038 |
| History of CVD^#^ | 1.11 (0.73-1.68) | p=0.64 |
| History of diabetes | 0.82 (0.53-1.26) | p=0.37 |
| History of hypertension | 0.76 (0.53-1.11) | p=0.16 |
| Kidneyfail group “AKI without CKD” | 0.88 (0.48-1.64) | p=0.69 |
| Kidneyfail group “CKD without AKI” | 1.61 (0.84-3.08) | p=0.15 |
| Kidneyfail group “AKI and CKD” | 0.74 (0.34-1.63) | p=0.46 |
| MELD spline 1^$^ | 0.94 (0.86-1.03) | p=0.20 |
| MELD spline 2^$^ | 1.22 (1.03-1.45) | p=0.021 |

*The change from day 1-3 was estimated as a slope from a linear model for each patient. Arginine concentration was modelled with log2.

^#^History of cardiovascular disease (CVD) were defined as history of heart failure, myocardial infarction, or stroke.

^$^The MELD score was modelled as a three-knot cubic spline.

HR = hazard ratio, CI = confidence interval, MELD = model for end-stage liver disease.

**Supplemental Table 36:** Arginine change day 1-3 and 30-day mortality in complete case sensitivity analysis, n = 340

|  | HR (95% CI) | P value |
| --- | --- | --- |
| Arginine change day 1-3* | 0.62 (0.27-1.46) | p=0.28 |
| Age | 1.03 (1.01-1.06) | p=0.002 |
| Sex, Male | 1.89 (1.20-2.97) | p=0.006 |
| History of CVD^#^ | 0.85 (0.50-1.42) | p=0.53 |
| History of diabetes | 0.77 (0.44-1.34) | p=0.35 |
| History of hypertension | 1.37 (0.86-2.18) | p=0.19 |
| Kidneyfail group “AKI without CKD” | 1.40 (0.57-3.43) | p=0.46 |
| Kidneyfail group “CKD without AKI” | 3.73 (1.81-7.65) | p<0.001 |
| Kidneyfail group “AKI and CKD” | 1.25 (0.41-3.75) | p=0.70 |
| MELD spline 1^$^ | 0.77 (0.69-0.86) | p<0.001 |
| MELD spline 2^$^ | 1.64 (1.37-1.97) | p<0.001 |

*The change from day 1-3 was estimated as a slope from a linear model for each patient. Arginine concentration was modelled with log2.

^#^History of cardiovascular disease (CVD) were defined as history of heart failure, myocardial infarction, or stroke.

^$^The MELD score was modelled as a three-knot cubic spline.

HR = hazard ratio, CI = confidence interval, MELD = model for end-stage liver disease.

**Supplemental Table 37:** Arginine change day 1-3 and 30-day mortality in last observation carried forward sensitivity analysis, n = 503

|  | HR (95% CI) | P value |
| --- | --- | --- |
| Arginine change day 1-3* | 0.77 (0.35-1.68) | p=0.51 |
| Age | 1.03 (1.02-1.05) | p<0.001 |
| Sex, Male | 1.48 (1.01-2.16) | p=0.043 |
| History of CVD^#^ | 1.05 (0.69-1.58) | p=0.83 |
| History of diabetes | 0.92 (0.60-1.41) | p=0.70 |
| History of hypertension | 0.79 (0.55-1.15) | p=0.22 |
| Kidneyfail group “AKI without CKD” | 0.98 (0.53-1.83) | p=0.96 |
| Kidneyfail group “CKD without AKI” | 1.63 (0.85-3.13) | p=0.14 |
| Kidneyfail group “AKI and CKD” | 0.66 (0.30-1.46) | p=0.30 |
| MELD spline 1^$^ | 0.97 (0.89-1.06) | p=0.54 |
| MELD spline 2^$^ | 1.14 (0.97-1.34) | p=0.10 |

*The change from day 1-3 was estimated as a slope from a linear model for each patient. Arginine concentration was modelled with log2.

^#^History of cardiovascular disease (CVD) were defined as history of heart failure, myocardial infarction, or stroke.

^$^The MELD score was modelled as a three-knot cubic spline.

HR = hazard ratio, CI = confidence interval, MELD = model for end-stage liver disease.

**Supplemental Table 38:** Homoarginine change day 1-3 and 30-day mortality in worst-case sensitivity analysis, n = 498

|  | HR (95% CI) | P value |
| --- | --- | --- |
| Homoarginine change day 1-3* | 0.17 (0.09-0.30) | p<0.001 |
| Age | 1.04 (1.02-1.06) | p<0.001 |
| Sex, Male | 1.38 (0.94-2.02) | p=0.10 |
| History of CVD^#^ | 1.16 (0.77-1.74) | p=0.49 |
| History of diabetes | 0.83 (0.53-1.29) | p=0.40 |
| History of hypertension | 0.77 (0.53-1.13) | p=0.18 |
| Kidneyfail group “AKI without CKD” | 0.83 (0.43-1.57) | p=0.56 |
| Kidneyfail group “CKD without AKI” | 1.68 (0.88-3.21) | p=0.12 |
| Kidneyfail group “AKI and CKD” | 0.91 (0.41-2.04) | p=0.82 |
| MELD spline 1^$^ | 0.94 (0.86-1.03) | p=0.20 |
| MELD spline 2^$^ | 1.20 (1.02-1.41) | p=0.032 |

*The change from day 1-3 was estimated as a slope from a linear model for each patient. Homoarginine concentration was modelled with log2.

^#^History of cardiovascular disease (CVD) were defined as history of heart failure, myocardial infarction, or stroke.

^$^The MELD score was modelled as a three-knot cubic spline.

HR = hazard ratio, CI = confidence interval, MELD = model for end-stage liver disease.

**Supplemental Table 39:** Homoarginine change day 1-3 and 30-day mortality in best-case sensitivity analysis, n = 498

|  | HR (95% CI) | P value |
| --- | --- | --- |
| Homoarginine change day 1-3* | 3.17 (1.74-5.75) | p<0.001 |
| Age | 1.03 (1.02-1.05) | p<0.001 |
| Sex, Male | 1.64 (1.12-2.40) | p=0.011 |
| History of CVD^#^ | 1.01 (0.66-1.54) | p=0.98 |
| History of diabetes | 0.83 (0.54-1.28) | p=0.41 |
| History of hypertension | 0.78 (0.54-1.13) | p=0.18 |
| Kidneyfail group “AKI without CKD” | 0.95 (0.51-1.77) | p=0.88 |
| Kidneyfail group “CKD without AKI” | 1.75 (0.91-3.35) | p=0.092 |
| Kidneyfail group “AKI and CKD” | 0.72 (0.32-1.59) | p=0.41 |
| MELD spline 1^$^ | 0.94 (0.86-1.03) | p=0.20 |
| MELD spline 2^$^ | 1.22 (1.03-1.45) | p=0.019 |

*The change from day 1-3 was estimated as a slope from a linear model for each patient. Homoarginine concentration was modelled with log2.

^#^History of cardiovascular disease (CVD) were defined as history of heart failure, myocardial infarction, or stroke.

^$^The MELD score was modelled as a three-knot cubic spline.

HR = hazard ratio, CI = confidence interval, MELD = model for end-stage liver disease.

**Supplemental Table 40:** Homoarginine change day 1-3 and 30-day mortality in complete case sensitivity analysis, n = 340

|  | HR (95% CI) | P value |
| --- | --- | --- |
| Homoarginine change day 1-3* | 0.25 (0.11-0.59) | p=0.001 |
| Age | 1.04 (1.02-1.06) | p=0.001 |
| Sex, Male | 1.81 (1.15-2.84) | p=0.010 |
| History of CVD^#^ | 0.83 (0.49-1.39) | p=0.47 |
| History of diabetes | 0.68 (0.38-1.21) | p=0.19 |
| History of hypertension | 1.33 (0.84-2.13) | p=0.23 |
| Kidneyfail group “AKI without CKD” | 1.22 (0.50-3.00) | p=0.66 |
| Kidneyfail group “CKD without AKI” | 3.95 (1.95-8.00) | p<0.001 |
| Kidneyfail group “AKI and CKD” | 1.34 (0.45-4.03) | p=0.60 |
| MELD spline 1^$^ | 0.76 (0.68-0.85) | p<0.001 |
| MELD spline 2^$^ | 1.69 (1.40-2.04 | p<0.001 |

*The change from day 1-3 was estimated as a slope from a linear model for each patient. Homoarginine concentration was modelled with log2.

^#^History of cardiovascular disease (CVD) were defined as history of heart failure, myocardial infarction, or stroke.

^$^The MELD score was modelled as a three-knot cubic spline.

HR = hazard ratio, CI = confidence interval, MELD = model for end-stage liver disease.

**Supplemental Table 41:** Homoarginine change day 1-3 and 30-day mortality in last observation carried forward sensitivity analysis, n = 503

|  | HR (95% CI) | P value |
| --- | --- | --- |
| Homoarginine change day 1-3* | 0.49 (0.20-1.16) | p=0.11 |
| Age | 1.03 (1.02-1.05) | p<0.001 |
| Sex, Male | 1.44 (0.98-2.11) | p=0.060 |
| History of CVD^#^ | 1.07 (0.71-1.61) | p=0.75 |
| History of diabetes | 0.91 (0.59-1.39) | p=0.65 |
| History of hypertension | 0.79 (0.54-1.14) | p=0.20 |
| Kidneyfail group “AKI without CKD” | 0.95 (0.51-1.78) | p=0.88 |
| Kidneyfail group “CKD without AKI” | 1.63 (0.85-3.13) | p=0.14 |
| Kidneyfail group “AKI and CKD” | 0.68 (0.30-1.51) | p=0.34 |
| MELD spline 1^$^ | 0.97 (0.88-1.06) | p=0.50 |
| MELD spline 2^$^ | 1.15 (0.98-1.35) | p=0.095 |

*The change from day 1-3 was estimated as a slope from a linear model for each patient. Homoarginine concentration was modelled with log2.

^#^History of cardiovascular disease (CVD) were defined as history of heart failure, myocardial infarction, or stroke.

^$^The MELD score was modelled as a three-knot cubic spline.

HR = hazard ratio, CI = confidence interval, MELD = model for end-stage liver disease.
