# Supplementary material for: Changes in nitric oxide inhibitors and mortality in critically ill patients: a cohort study
Source: Ann Intensive Care. 2024 Aug 27;14:133. doi: 10.1186/s13613-024-01362-7 (PMC11349968; doi:10.1186/s13613-024-01362-7)
Supplement: Supplementary file 7 — Supplementary Material 7 [file 13613_2024_1362_MOESM7_ESM.docx]

**Additional File 7:** Correlation

**Supplemental Figure 15**: Correlation between change in ADMA concentration day 1-3 and ADMA concentration at admission.


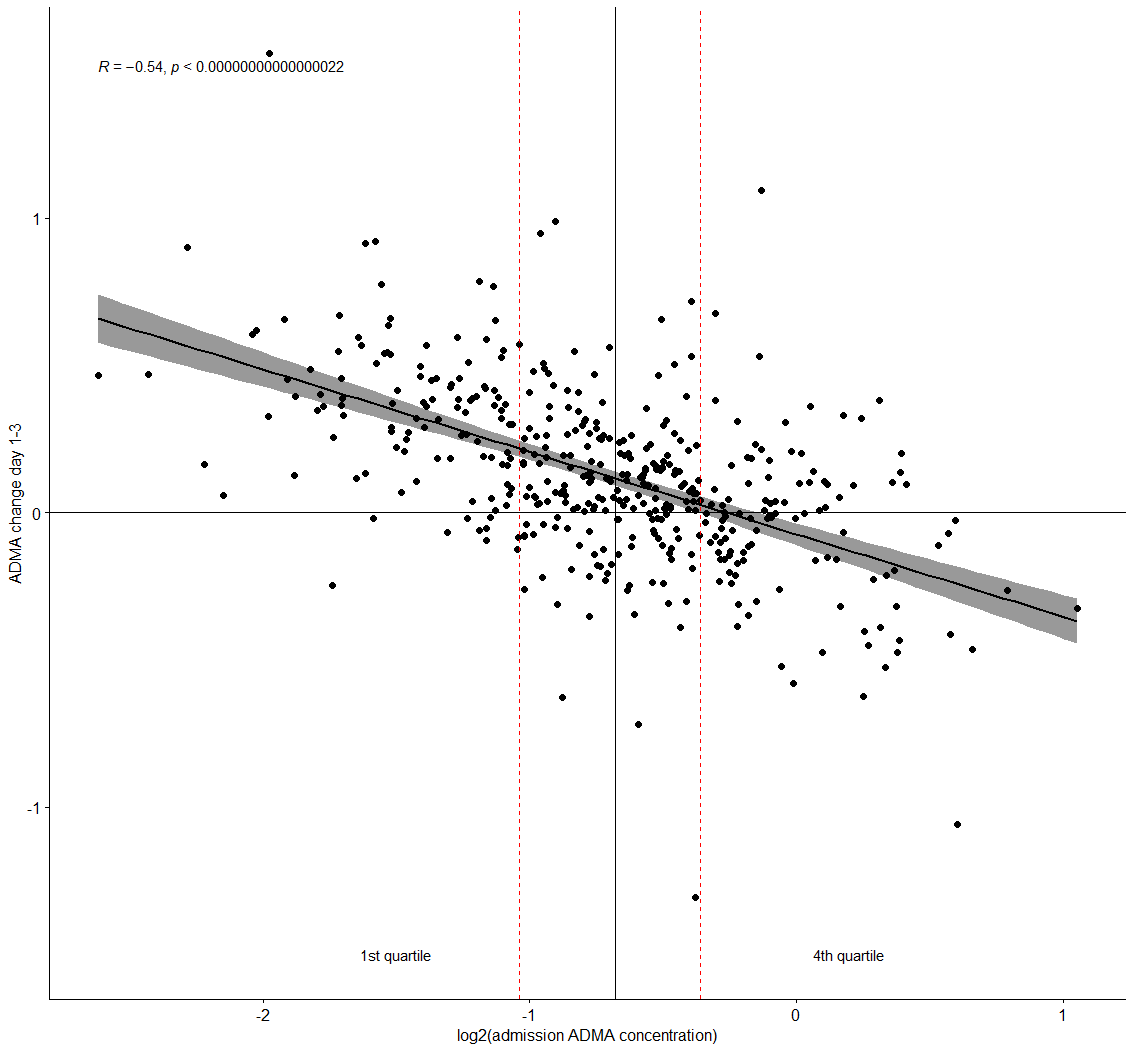


The change from days 1-3 was estimated as a slope from a linear model for each patient. The black horizontal line indicates a slope of zero (no change in concentration days 1-3). The black vertical line indicates the median ADMA admission concentration. The dashed red vertical lines indicate the interquartile range. ADMA = asymmetric dimethylarginine.
